# New constraints on Cenozoic subduction between India and Tibet

**DOI:** 10.1038/s41467-023-37615-5

**Published:** 2023-04-07

**Authors:** Liang Liu, Lijun Liu, Jason. P. Morgan, Yi-Gang Xu, Ling Chen

**Affiliations:** 1 State Key Laboratory of Isotope Geochemistry and CAS center of Excellence in Deep Earth Science, Guangzhou Institute of Geochemistry, Chinese Academy of Science, Guangzhou, 510640 China; 2 Department of Geology, University of Illinois at Urbana-Champaign, Urbana, IL 61801 USA; 3 Southern Marine Science and Engineering Guangdong Laboratory (Guangzhou), Guangzhou, 511458 China; 4 Department of Marine Science and Engineering, Southern University of Science and Technology, Shenzhen, 518055 Guangdong China; 5 State Key Laboratory of Lithospheric Evolution, Institute of Geology and Geophysics, Chinese Academy of Sciences, Beijing, China

**Keywords:** Geodynamics, Geophysics, Structural geology

## Abstract

The type of lithosphere subducted between India and Tibet since the Paleocene remains controversial; it has been suggested to be either entirely continental, oceanic, or a mixture of the two. As the subduction history of this lost lithosphere strongly shaped Tibetan intraplate tectonism, we attempt to further constrain its nature and density structure with numerical models that aim to reproduce the observed history of magmatism and crustal thickening in addition to present-day plateau properties between 83°E and 88°E. By matching time-evolving geological patterns, here we show that Tibetan tectonism away from the Himalayan syntaxis is consistent with the initial indentation of a craton-like terrane at 55 ± 5 Ma, followed by a buoyant tectonic plate with a thin crust, e.g., a broad continental margin (Himalandia). This new geodynamic scenario can explain the seemingly contradictory observations that had led to competing hypotheses like the subduction of Greater India versus largely oceanic subduction prior to Indian indentation.

## Abstract (web summary)

By evaluating model predictions with multiple geological data, the study shows that Tibetan tectonism is most consistent with the initial indentation of a cratonic terrane, followed by subduction of a buoyant tectonic plate resembling a continental margin.

## Introduction

**Fig. 1:**
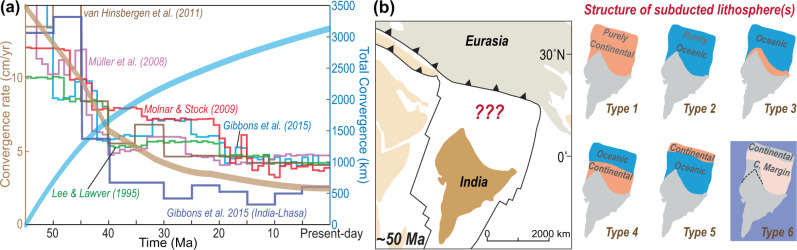
Proposed structures for the subducted lithosphere that was between India and Tibet in the early Eocene. **a** The convergence history between India and Eurasia in different plate reconstructions^11–15^. The thick brown line illustrates the average convergence rate of these different studies, and the thick blue line shows the corresponding total convergence. **b** Early Paleogene position of the Indian Subcontinent with different hypotheses (or model types) for the nature and structure of the ‘lost’ lithosphere between India and Tibet^1,2,4,11,17,20–22^. Based on the numerical modeling in this study (also in Liu et al., 2021a^44^), the external terrane was also part of the Indian Subcontinent (Type 6). We propose a wedge-shaped continental margin between the terrane and Indian Subcontinent, based on the recent recognition that the hard collision happened ~20 Myr earlier in regions towards the western Himalayan syntaxis than in eastern regions^22^. C. Margin- Continental Margin. Type 2, 3, and 4 models are inconsistent with the geological record but help to demonstrate the effects of various initial conditions on model evolution (more discussion in the main text).

While researchers agree that the Tibetan Plateau largely formed during the Cenozoic collision of India with Eurasia, the structure and property of the subducted plate between the two remain heavily debated^1–7^. Paleomagnetic studies reveal that the Indian Subcontinent was ~3000 km south of its present-day location during the Paleocene (Fig. 1a)^1–4,8–15^. Given that 1000–2000 km of ~north-south convergence has been accommodated by crustal shortening within the Himalayas and Asia^1,3^, a 1000–2000 km long tectonic plate should have therefore existed between India and Eurasia before the Eocene (Fig. 1b). Some authors suggest that this ‘lost’ plate consisted entirely of Indian-type continental lithosphere (Greater India, i.e., Type 1 in Fig. 1b) that initially abutted Australia^16^, with fluvial drainage starting to connect Eurasian highlands to Himalayan foreland basins no later than 40 Ma^17–19^. Alternative hypotheses consider this subducted lithosphere to have contained either a young backarc oceanic basin that formed to the north of an intra-oceanic subduction zone (Types 2-3 in Fig. 1b)^4,11,20,21^, or an Atlantic-type oceanic basin with old seafloors formed during the Cretaceous as the Greater-Tethyan Himalayas rifted from the Indian Subcontinent (Greater Indian Basin, i.e., Types 4–5 in Fig. 1b)^1,2,22^, in order to explain the limited volume of accreted continental crust that is exposed at the surface^2,5,23^.

Many reconstruction models have been proposed to explain the structure and properties of the subducted plate^6,11,24–27^, with consensus yet to be reached. Multiple models utilize seismic tomography as a potential constraint^11,26,27^, which, according to a recent review^6^, cannot effectively discriminate in situ between the different proposed properties of the subducted blocks.

**Fig. 2:**
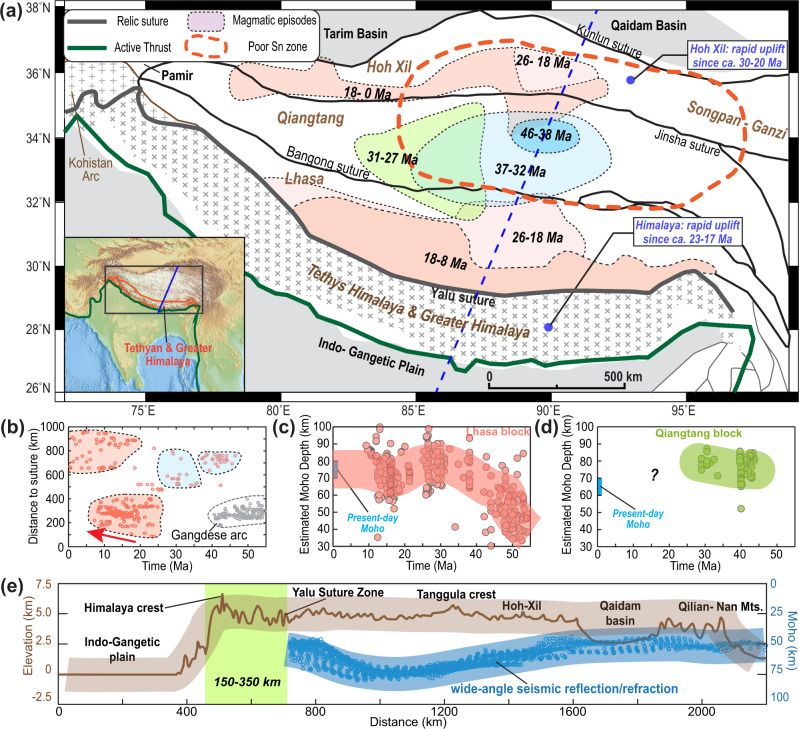
Magmatic, geochemical, and geophysical constraints on Tibetan Plateau evolution. **a** A sketch of major terrane elements of the Tibet Plateau and Himalayas, with colored patterns representing magmatism^4,36,46,54^. The dashed blue line marks the model location in Fig. 3. **b** History of Tibetan magmatism along the north-south direction^31,38^. Distance denotes the distance between the sampling location of Moho depth and the suture zone. **c**, **d** Crustal thickness evolution of Lhasa and Qiangtang based on magmatic geochemistry^79^. **e** Present Tibetan topography (brown) and Moho depth (blue), e.g., along the transect in Panel **a**^4,80,82^. Moho depth is from Zhang et al., 2014, which has data coverage over the range of the on-plateau magmatism (also see^82^). The light green box marks the range of the accreted crust before the indentation of the Indian Subcontinent, e.g., the Tethyan and Greater Himalaya sequences^2,5^. In panels **c**–**e**, the smooth-shaded envelopes approximate the overall mean trends of data constraints.

Geodynamically, the subduction of continental and oceanic lithosphere, due to their contrasting buoyancy structures, should affect the overriding plate in fundamentally different ways^28–30^. Therefore, the unique intraplate tectonics of Tibet (Fig. 2) may contain critical information on the nature of the lost plate during the India-Asia collision. It is now generally accepted that in the regions away from the Himalayan syntaxis, post-50 Ma intraplate magmatism (Fig. 2a, b), crustal shortening (Fig. 2c, d), and/or surface uplift (Fig. 2a) all commenced initially within the central plateau and then migrated/jumped both southward and northward^4,23,31–40^.

Various scenarios have been proposed to explain the above intraplate tectonics, especially the on-plateau magmatism. These include lithospheric delamination^23,41–43^, flat subduction^2,4^, slab tearing and/or roll-back^2,23,24,31^, and subduction-related mantle upwelling^34^. Previously these proposed scenarios could not be sufficiently evaluated using prior constraints like plate reconstructions and seismic tomography^6^, while a quantitative model that simultaneously considered the dynamics of plate subduction from the south and the resulting plateau-scale records (Fig. 2) was lacking. A recent geodynamic study^24^ that attempted to do this assumed a ~3000 km of upper plate shortening, placing the southern Tibetan margin at 11 °N around Paleocene, with Indo-Asian convergence mostly reflecting the Asian lithosphere underthrusting below Tibet. However, many previous paleomagnetic studies indicate that the southern Tibetan margin was around 20 °N in the Paleocene^4,6^, requiring significantly more (~1000 km) plate subduction beneath the southern Tibetan margin than these workers had assumed^24^. In addition, that study’s modeled Qiangtang and Hoh-Xil lithosphere appears too cold (lithosphere >150 km thick and Moho temperature <700 °C) to permit melting to happen at ~40–30 Ma and <20 Ma, respectively, in contrast to the observed Tibetan magmatic history (Fig. 2a, b).

Here we have revisited this problem with updated plate kinematics (Fig. 1a) and the incorporation of additional time-space constraints on Tibetan magmatism. Based on these observations and a suite of differing 2-D geodynamic evolution models, we will evaluate the quantitative relationships between various proposed subduction scenarios (Fig. 1b) and the N-S characteristics of intra-plateau processes (Fig. 2). This model-based analysis indicates that the lost lithosphere directly north of the Indian Subcontinent was most likely a thinned continental margin that contained little to no oceanic crust.

## Results

### Numerical quantification of subduction-to-surface relationships

**Fig. 3:**
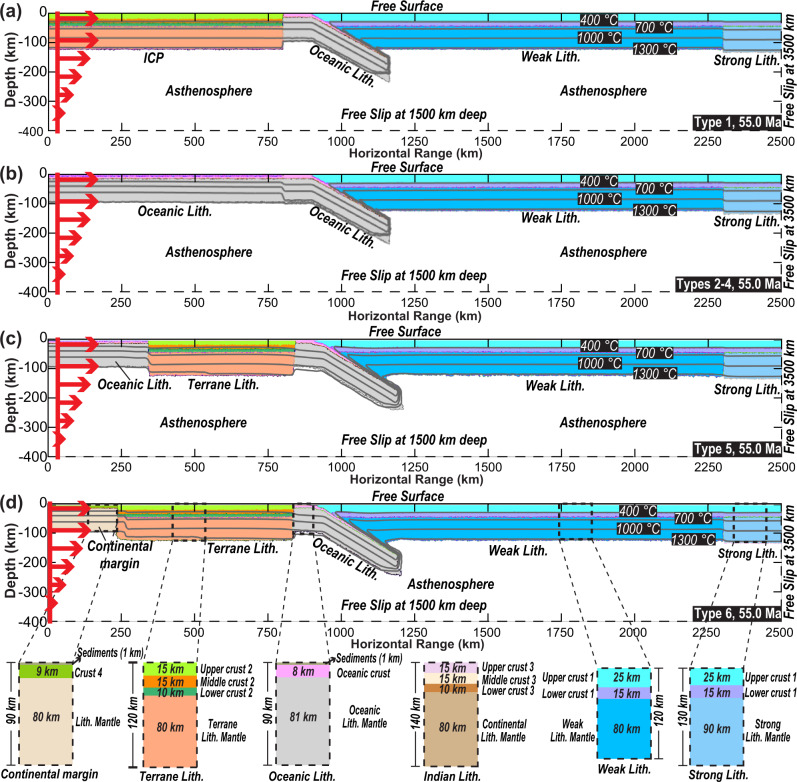
Initial and boundary conditions for the numerical models. **a** Type 1 models (Runs 1–4, Supplementary Figs. S5 and S6), where the incoming plate before the Indian Subcontinent indentation is purely continental. ICP-Incoming Continental Plate (i.e., Greater India). Strong Litho.- i.e., Asian interiors. Weak Litho.- i.e., Tibetan terranes. **b** Type 2 models (Runs 5–6, Supplementary Figs. S7 and S8), where the incoming plate north of the Indian Subcontinent is purely oceanic (i.e., Greater Indian Basin). The ocean has an assumed age of 40 Myr^1^. Type 3 (Run 7–10, Supplementary Figs. S9 and S10) and Type 4 (Run 11–14, Supplementary Figs. S11 and S12) models share the same initial condition as Type 2, except that there is an additional 200 km or 900 km long ICP between the oceanic segment and Indian Subcontinent in Type 3 and type 4 models, respectively (Fig. 1b)^4,21^. **c** Type 5 models (Runs 15–22, Supplementary Figs. S13–S16), where a preceding terrane separates with the Indian Subcontinent by an 80 Myr oceanic block. The terrane Lithosphere is 500 km long in Runs 15–18 and 600 km long in Runs 19–22 (Supplementary Table S2). The compositional density of the ocean lithospheric mantle is 3.39 g/cm^3^, and that of the terrane lithospheric mantle is 3.37 g/cm^3^. **d** Type 6 models (Runs 23–40, Fig. 5, Supplementary Figs. S17–S20) (Supplementary Table S2). These are similar to Type 5 but with a more buoyant lithosphere (compositional density anomaly of the lithospheric mantle −0.02 g/cm^3^ and the lithospheric thermal age −40 Myr) behind a more buoyant terrane (the compositional density anomaly of its lithospheric mantle is −0.03 g/cm^3^). This portion of the plate has a thin (~10 km) crust as required by the Himalayan crustal volume^44^ and thus more resembles a thinned continental margin. The terrane length in the Type 6 models is ~600 km long^44^. The enlarged views demonstrate the material distribution in each lithospheric domain.

To quantitatively reproduce the corresponding subduction-to-surface relationships of different tectonic scenarios (Supplementary Fig. 1b), we designed 2D numerical models with an adaptive finite-element mesh using a free surface (Fig. 3, Supplementary Tables S1 and S2 in the supplements), with other boundaries left free to slip. All materials in the numerical models have a viscoplastic rheology, with a Drucker–Prager yield criterion used to determine the visco-plastic transition (Supplementary Figs. S1–S3). During subduction and collision, mafic crustal materials experience an Arrhenius-type eclogite phase transformation, and in the mantle transition zone, mantle materials experience deeper phase transformations from olivine to wadsleyite and ringwoodite to bridgmanite (Supplementary Fig. S4). When modeling the behavior of multi-phase melting, the effects of melting degree and source mineral assemblages (crust and asthenospheric or lithospheric mantle) on the solidus are also considered—see Methods for further information and specific rheological details.

We utilize intra-plateau records between 83°E and 88°E (Fig. 2) as the primary constraints for these models, which focus on exploring the tectonic evolution along a southwest-to-northeast transect (i.e., the blue dashed line in Fig. 2a). We use the southern, central, and northern plateaus to roughly represent the Lhasa, Qiangtang, and Hoh-Xil terranes. The modeled relic crust from the accreted terrane and/or Greater India approximates observed Himalayan sequences^2,4^. Finally, the present-day plateau morphology, the histories of intra-plateau magmatism and crustal thickness across the plateau, and the predicted present-day upper mantle structure are evaluated against their corresponding observations. In the models discussed here, the total convergence since 55 Ma is ~3150 km (Fig. 1a), accommodated by subduction of the plate north of the Indian Subcontinent (~2000 km), Asian crustal shortening (~600–1000 km), and Indian Subcontinent indentation (~150–550 km) (Fig. 3). Given the trade-off between the rate of convergence and the timing of tectonic events (Fig. 1a) and our 2D idealization of the convergence process, these numerical models emphasize the importance of sequential geodynamic processes rather than their absolute timing. For the observed crustal thickening, 2D models tend to underestimate the amount of shortening due to their omission of lateral extrusion. Therefore, these experiments provide minimum estimates of shortening-related thickening.

**Fig. 4:**
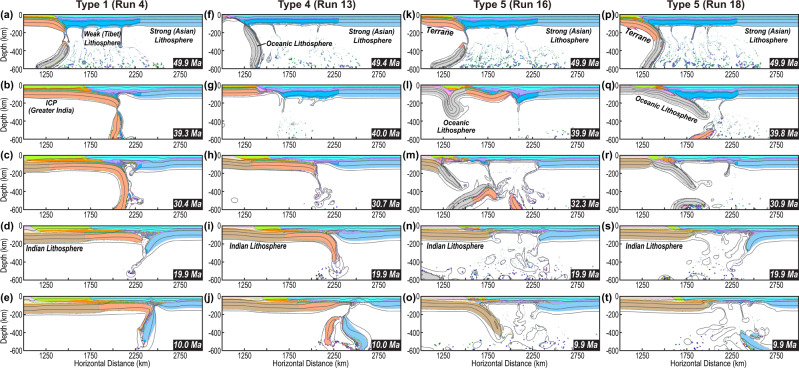
Typical geodynamic evolution seen in numerical models. In Type 1 models (Fig. 3a): **a**, **b** flat subduction develops once the former oceanic slab separates with the following continental block. **(c)** the flat slab bends downward when it collides with the strong overriding plate, and later, its deeply subducted portion breaks up with the shallow one. **d**, **e** the remaining flat slab bulldozes the lower lithosphere (below crust) of the strong overriding plate into the deep mantle. In **Type 4** models: **f** the oceanic portion develops steep subduction; and **g–j** the following model evolution is largely like that of Type 1. In **Type 5** models (Fig. 3c, Table S2): **k**–**o** when the overriding plate is weak, flat terrane subduction happens first, followed by steeper ocean subduction; **p**–**t** when the overriding plate is strong, steep subduction of both the terrane and oceanic portion persists until the Indian indentation. More descriptions and presentations for modeling results can be found in the main text, supplements, and supplementary movies.

**Fig. 5:**
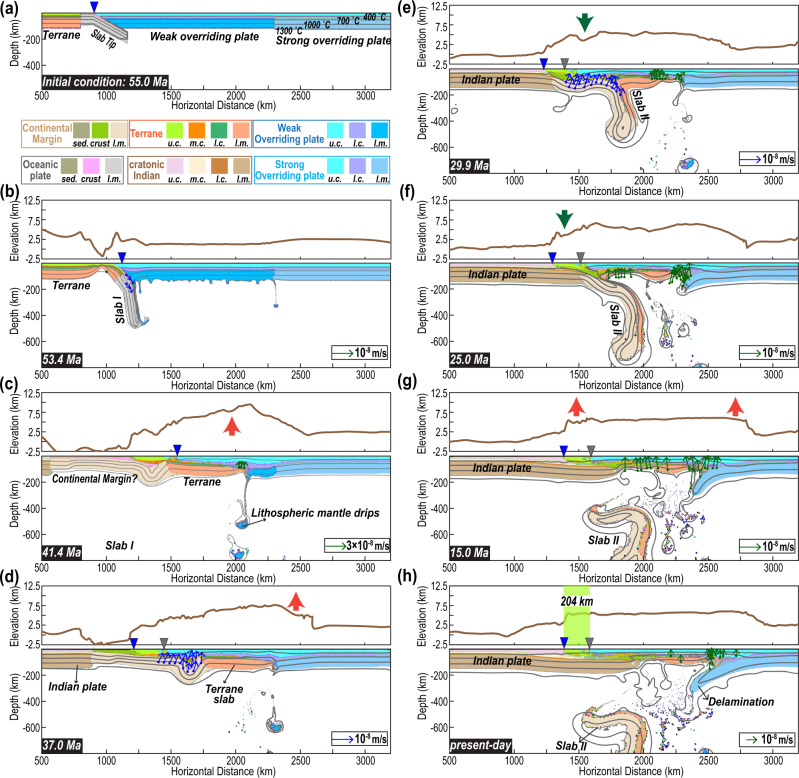
Cenozoic subduction and Tibetan evolution. **a**–**h** Snapshots of the modeled material field and surface topography. The material figures: blue triangles mark the active trench, and grey triangles mark the abandoned ones; Green arrows demonstrate the melting locations, while the blue ones present the locations for oceanic slab dehydration. The red and green bold arrows in the topography figures demonstrate the locations where uplift and subsidence happen, respectively. The light green box in **h** marks the range of accreted crust before the indentation of the Indian Subcontinent. The melting/fluid tracers are sampled every 50 km, and their velocities are from Eqs. S16-S17 in **Methods**. The modeled histories of deviatoric stress, viscosity, density, and strain rate for the best-fit Type 6 model are shown in Figures S1-S4, with additional sensitivity tests on the robustness of model results in Figures S17–S20 and Liu et al., 2021a. More discussion on the differences between Type 5 and 6 models is in the supplements. u.c.-upper crust, m.c.-middle crust, l.c.-lower crust, l.m.-lithospheric mantle, sed.-sediments.

We first designed five models that acknowledge the key tectonic components and their contrasting subduction styles in the literature (Type 1–5 models) (Figs. 1b and 3, Supplementary Table S2). Among these, models with a broad continent or young ocean basin (Types 1–2) could result in widespread flat slabs due to the relatively buoyant subducting plate (Figs. 4a–e, Supplementary Figs. S5–S8, Supplementary Movies 1 and 2)^30,44^. In Types 3–4 models (Fig. 1b), flat subduction of the continental block (Greater India) happens after the steeper subduction of the preceding oceanic block (Fig. 4f–j, Supplementary Figs. S9–S12, Supplementary Movies 3 and 4). Instead, in Type 5 models where the oceanic block subducts after the continental one (Fig. 3c), two types of model evolution are observed: (1) when the overriding plate is weak enough (i.e., the maximum yielding stress is ≤150 MPa, Supplementary Table S2), this allows flat subduction of the continental terrane (Fig. 4i); then, due to the buoyancy contrast between the terrane and its following denser block, a new subducting slab forms below south-central Tibet which pulls the flat terrane slab into the deep mantle^30^ (Fig. 4m, Supplementary Fig. S13–S16, Supplementary Movie 5); and (2) when the overriding plate is strong enough to resist flat terrane subduction (i.e., the maximum yielding stress ≥150 Ma, Supplementary Table S2), steep subduction sustains until the Indian indentation (Fig. 4p–t, Supplementary Figs. S13–S16, Supplementary Movie 6)^28^. In addition, because we adopt relatively weak Tibetan terranes in most models (Fig. 3) following previous studies^4,31,42,43^, the overriding plate tends to experience drip-like delamination during the collision process (e.g., Fig. 5c, Supplementary Movies 1, 4, 5, and 7). Tibetan lithosphere delamination and/or flat slab detachment can lead to direct contact between the thickened Asian crust and the hot asthenosphere, which facilitates melting. After this, the lower Asian lithosphere gradually develops peeling-like delamination or sub-crustal subduction along the above thickened hot crust^28,44,45^, during which a double subduction scenario can occasionally develop (e.g., Figs. 4o, s and 5f, Supplementary Fig. S1d). See Supplementary Figs. S5–S16 and Supplementary Movies 1–6 for the evolution of Type 1–5 models.

### Evolution of the representative numerical model

**Fig. 6:**
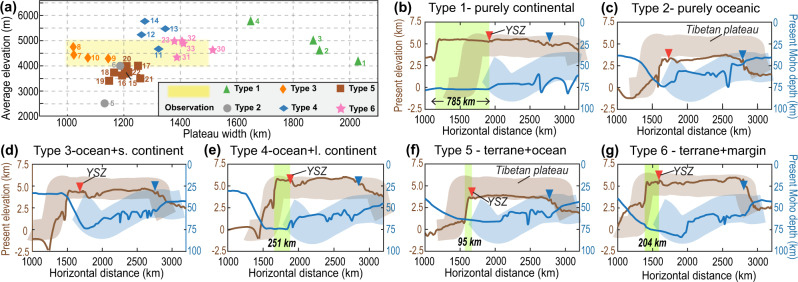
Present-day plateau morphology. **a** Plateau width (perpendicular to trench) and mean elevation. Numbers close to symbols denote different numerical models (Table S2). **b**–**g** Plateau topography and Moho depth in Type 1 (Run 3, Figures S5c, S6c), Type 2 (Run 6, Figures S7b, S8b), Type 3 (Run 9, Figures S9c, S10c), Type 4 (Run 13, Figures S11c, S12c), Type 5 (Run 18, Figures S13d, S14d), and Type 6 (Run 23, Fig. 5) models, respectively. The thick background shadings illustrate the topography and Moho distribution of the Tibetan Plateau (Fig. 2e). The light green box marks the range of the accreted crust before the indentation of the Indian Subcontinent, i.e., the Himalayas. The horizontal range of the numerical models (i.e., along the blue dashed line in Fig. 2a) between the YSZ (red triangle) and the location beyond which the topography rapidly drops (blue triangle) is evenly divided into three portions: southern, middle, and northern plateau. s.-small. l.-large.

**Fig. 7:**
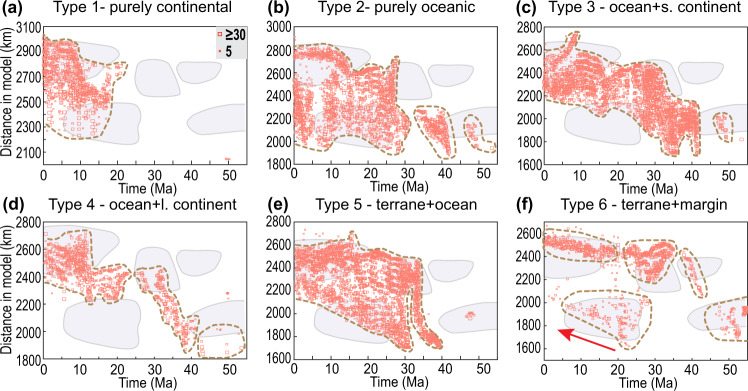
Magmatic history in different numerical models. **a**–**f** Magmatic history in Type 1 (Run 3, Figures S5c, S6c), Type 2 (Run 6, Figures S7b, S8b), Type 3 (Run 9, Figures S9c, S10c), Type 4 (Run 13, Figures S11c, S12c), Type 5 (Run 18, Figures S13d, S14d), and Type 6 (Run 23, Fig. 4) models, respectively. Background shadings illustrate the time-space range of observed magmatism (Figs. 2a and 2b); the modeled melts (see **Methods**) are projected to their present-day locations and are shown for every 50 km along the horizontal direction with the symbol size representing the amount of melting. s.-small. l.-large.

**Fig. 8:**
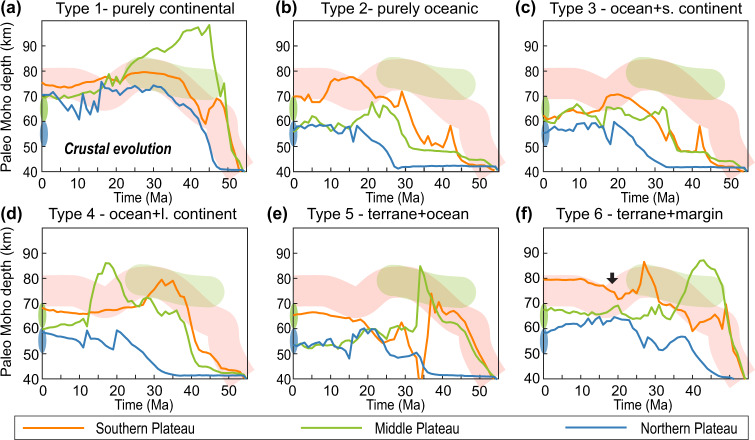
Crustal deformation in different numerical models. **a–f** Evolution of crustal thickness in Type 1 (Run 3, Figures S5c, S6c), Type 2 (Run 6, Figures S7b, S8b), Type 3 (Run 9, Figures S9c, S10c), Type 4 (Run 13, Figures S11c, S12c), Type 5 (Run 18, Figures S13d, S14d), and Type 6 (Run 23, Fig. 5) models, respectively. Background thick shadings are constraints from Figs. 2c and 2d. The horizontal range of the numerical models (i.e., along the blue dashed line in Fig. 2a) between the YSZ (red triangles in Fig. 6) and the location beyond which the topography rapidly drops (blue triangles in Fig. 6) is evenly divided into three portions: southern, middle, and northern plateau. S.-small. L.-large.

**Fig. 9:**
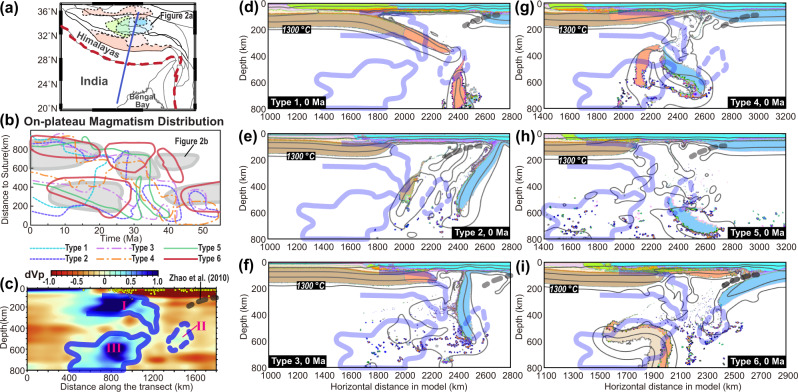
Comparisons between model results and geological and geophysical observations. **a** The distribution of magmatism on the Tibetan Plateau (Fig. 2a) and the transect location (blue line) for the seismic tomography result in Panel **c**. **b** Magmatic history in Type 1 (Run 3), Type 2 (Run 6), Type 3 (Run 9), Type 4 (Run 13), Type 5 (Run 18), and Type 6 (Run 23) models, respectively. Background shadings illustrate the time-space range of on-plateau magmatism (see Fig. 2a, b). A more detailed presentation of this comparison is also shown in Fig. 7. **c** The P-wave seismic tomography cross-section of the upper mantle beneath North India and Tibet Plateau (after Li et al. 2008)^81^. The potential upper surface of the subducted/delaminated Asian lithosphere (black dashed line) is based on the results from P and S receiver functions^46^. More seismic tomography results along different transects are shown in Supplementary Fig. S22. **d**–**i** the “present-day” snapshots in Runs 4, 6, 9, 13, 18, and 23, respectively. Contours of high-seismic-wave-speed anomalies below Tibet are from Panel **c**. Note that the slab-like structures in **e**, **f**, and **h** are delaminated/delaminating Strong Lithosphere, i.e., Asian lithosphere.

**Fig. 10:**
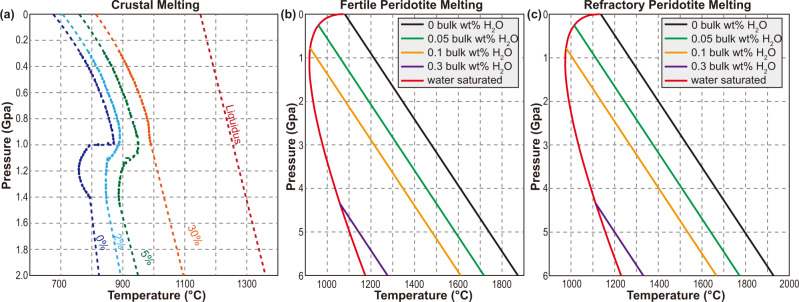
Solidi for the crustal and mantle materials^56,62^. **a** The solidus for the crustal materials is based on lab experiments^73^. 2% means that amphibolite has experienced 2% melting. Liquidus equals 100% melting degree. **b**, **c** The solidus for the mantle materials is based on Morgan (2001) and Katz et al. (2003). “0.1 bulk wt. %“ refers to the water content we use for calculating the solidus. The fertile peridotite solidus is used for simulating the melting of the asthenospheric mantle and the weak overriding-plate lithospheric mantle (i.e., the metasomatized Tibetan lithospheric mantle)^31,34,38^. The refractory mantle solidus is used for calculating the melting of other mantle materials.

In addition to the first five models of the lost lithospheres that are based on different lines of observations (Fig. 1b), we also consider the Type 6 model (Figs. 3d, 5), which appears to better fit the morphology of the present plateau and the history of Tibetan intraplate tectonism (Figs. 6–9). Compared to the models in Liu et al., 2021a^44^, here we focus on simulating the surface responses of subduction. Therefore, we explicitly modeled the melting behaviors of different mantle and crustal materials (Figs. 5, 10, 7). Furthermore, as this model includes most of the typical phenomena in other models (Fig. 4), e.g., slab tearing, flat subduction, drip-like delamination, slab rollback, and Asian lithosphere subduction/delamination, we use it to introduce the simulated subduction-to-surface relationships. In the following, we describe the three main stages seen in the evolution of the Type 6 model (Run 23). More sensitivity tests regarding this model are given in the supplement (Figures S1–S4 and S17–S20).

Stage I: Early Eocene terrane underthrusting. Because of its large buoyancy contrast with the following terrane, the initial oceanic slab tears and quickly sinks into the mantle (Fig. 5a, b)^30^. This event would induce melting resembling magmas seen in the Gangdese Arc (Fig. 7f)^31^.

Due to the continuous convergence, the lower terrane lithosphere decouples from its upper crust along a weak middle crust (Fig. 5c)^25,28,30,44^. The resulting flat terrane slab shortens the Tibetan lithosphere and induces its delamination (Fig. 5c), which can explain the ca. 45-30 Ma magmatism in central Tibet and the present-day poor Sn propagation there that also implies the current absence of strong lithosphere (Figs. 2a, b and 7f)^4,31,46^. This lithosphere removal and crustal shortening led to a broad uplift event (Fig. 5c)^23,35,37^. We emphasize that a limitation of these regional models, i.e., the dynamic subsidence of previously subducted Tethyan slabs, is not simulated within an even longer model history (e.g., Supplementary Fig. S21)^12,47^. This makes the modeled absolute elevation values less meaningful than the relative spatial topographical changes that we highlight here.

Stage II: Thinned continental margin subduction. Following the initially underthrust terrane, the down-going plate has a thin (~10 km) crust and continent-like mantle lithosphere characteristic of a thinned continental margin. The lack of a thick crust reduces upper plate compression. Therefore, the thickened upper crust (Fig. 5c) can slide above the post-delamination heated lower crust (Fig. 5d, Supplementary Fig. S1)^48,49^. Consequently, the middle-northern plateau becomes flatter than before (Fig. 5d). This model result helps to explain the low post-Early Eocene erosion rates in the central plateau^50^ and the slight changes in the topography of Qiangtang after the major lithospheric delamination event implied by magmatic geochemistry^51^.

The greater density of the continental-margin slab than that of its preceding terrane slab (Supplementary Table S1) tends to trigger instability in their transitional region (Fig. 5d). As a result, when the tip of the flat terrane slab finally collides with the strong Asian lithosphere, the resulting enhanced resistance finally leads to the formation of a new subducting slab below south-central Tibet (Figs. 4i and 5e). This subduction behavior occurs whenever the lithospheric mantle of the continental-margin slab is denser (~0.02 g/cm^3^) than that of the terrane^44^. Furthermore, the sinking continental-margin slab pulls the flat terrane toward the trench, generating transient subsidence as seen in southern Lhasa (Fig. 5e), which can possibly explain the formation of the Kailas basin^52^. Given that additional dynamic topography sources from even earlier subduction that are not considered here could have further decreased the elevation of southern Tibet^47,53^, the Kailas and/or Lhasa regions could have also been at a low elevation state (e.g., <2 km) similar to the previous inferences^23,52,54^.

The retreating continental-margin slab then tears the terrane slab apart (Fig. 5f), while the northern segment remains coupled to the base of Tibet (Fig. 5g, h). The reestablished magmatism above the slab is consistent with the observed on-plateau magmatic history (Figs. 5f and 7f). In addition, the lateral density and viscosity contrasts cause the Asian lower lithosphere to peel away (Fig. 5f)^42,55,56^. These processes, together with the relic terrane underplating the central overriding plate, can explain the observed double magmatic belts along the two ends of the plateau with a post-25 Ma magmatic hiatus in the center (Fig. 2b)^4,31,34,38^.

Stage III: Indian Subcontinent indentation. The simultaneous slab rollback and foundering of the Asian lithosphere will raise the elevation of the Himalaya and Hoh-Xil, respectively (Fig. 5f, g)^4,33,36,54,57^. The India Subcontinent indentation causes the former slab to tear off (Fig. 5g) and terminates Lhasa magmatism^4,31^, consistent with observations (Figs. 2b, 5h, and 7f). Since then, sparse mantle melting that induces surface magmatism mainly occurs within the northern plateau (Figs. 5h and 7f).

### Distinct tectonic responses of the different subduction scenarios

To distinguish between the different model scenarios, we compare their predictions for present plateau morphology, magmatic history, crustal thickness evolution, and upper mantle structures (Figs. 6–9).

We first compare the modeled and observed “present-day” plateau width and Moho depth, as shown in Fig. 6. The Type 1 models (purely continental subduction) generate the broadest plateau and Himalayan orogen, both larger than observed (Fig. 6a, b). The resulting present-day Moho is also thicker and flatter (Fig. 6b), suggesting an excessive amount of continental accretion^44^. The purely oceanic subduction in Type 2 models induces limited compression within the overriding plate; thus, the “present-day” plateau is lower than observed (Fig. 6a, c). In the Type 3 models, the 200 km long “Greater India” generates a higher but narrower plateau than those in the Type 2 models (Fig. 6a). However, the 200 km long Greater India only leaves a 0–50 km wide accreted crust after subduction (Fig. 6d). The Type 4 models, in which the “Greater India” is ~900 km long following a ~1100 km long oceanic segment, form a slightly higher but narrower plateau than observed (Fig. 6a, e), which suggests an excessive amount of continental collision like that in the Type 1 models. The Type 5 models, where 1400–1500 km long ocean subduction follows 600–500 km long terrane underthrusting, create a reasonably wide plateau and Himalayas, and their stepwise Moho distribution resembles observations (Fig. 6a, f). However, the dense oceanic slab in Type 5 models pulls the topography down, leading to a final plateau that is only ~3.5-4 km high (Fig. 6f). Finally, in the Type 6 model, where a buoyant continental margin instead of oceanic lithosphere subducts following the terrane, the present plateau elevation and width best match this suite of observations (Fig. 6g).

In addition to the present-day plateau structures, the predicted magmatic history (Fig. 2a, b), an observational record rarely used in previous geodynamic studies, also differs widely between models (Fig. 7). Type 1 models, due to widespread flat subduction (Supplementary Figs. S5 and S6, Supplementary Movie 1), inhibit melting from occurring until very late, when the Asian-interior (strong) lithosphere starts to delaminate in response to the indentation of the Indian Subcontinent (Fig. 7a). In the models where oceanic subduction dominates continental subduction (Types 2 and 3), the oceanic slab entrains the relic terrane and the overriding lithosphere, leaving a broad mantle wedge (Supplementary Figs. S7–S10, Supplementary Movies 2 and 3). Melting has been temporally continuous on the south above slabs and further extends northward across the entire plateau after ~25 Ma when Indian Subcontinent flat subduction triggers upper plate delamination (Fig. 7b, c). In Type 4 models, episodic melting happens (Fig. 7d). The first episode (>40 Ma) corresponds to the preceding oceanic subduction and slab tearing, the second (30–40 Ma) to the delamination of the weak Tibetan lithosphere, and the last (<20 Ma) to the removal of the Asian lithosphere (Supplementary Figs. S11 and S12, Supplementary Movie 4). Melting stops in the south-central plateau ~after 30 Ma when the Indian Subcontinent indentation shuts down its mantle wedge (Supplementary Figs. S11 and S12). The magmatic history after 40 Ma in the Type 5 models (Fig. 7e) is similar to those in Types 2 and 3. Before 40 Ma, terrane underthrusting prevents melting within the mantle wedge (e.g., Fig. 4l, 4p, also see Supplementary Figs. S13 and S16). Type 6 models (Supplementary Movies 7 and 8) display a melting history with the most time-space variations, including melting associated with early oceanic subduction (>50 Ma), Tibetan lithosphere delamination caused by the advancing terrane flat slab (20–40 Ma), post-terrane continental-margin subduction and slab rollback (20–30 Ma), and Asian lithosphere removal (<20 Ma). These magmas sweep over the entire plateau, following a pattern that most closely resembles the observed one (compare Fig. 2b vs. Fig. 7f).

We can also compare the modeled crustal thickening histories in different scenarios with available observational constraints. In Type 1 models, the crustal thickness increases rapidly after the initial collision, earlier than observed. In contrast, Types 2 and 3 models with dominantly oceanic subduction increase the crustal thickness only after continental subduction starts, too late when compared to observations. The quasi-linear relationship between the timing of continental subduction and crustal thickening (Fig. 8) suggests that the incoming plate north of India should be more buoyant than a purely oceanic plate. However, it cannot be entirely continental, as this would over-predict paleo-crustal thicknesses. The Type 4 models, which have an intermediate age of continental subduction, still under-predict the timing of the initial crustal thickening (Fig. 8d). This systematic delay in model predictions supports Type 5-6 models where a buoyant terrane subducts earlier than that in Type 4 models. Compared to Type 5 models (Fig. 8e), the Type 6 models better fit paleo-crustal thickness proxies (Fig. 8f), thus representing the preferred tectonic scenario.

Finally, we compare the modeled present-day structure of the upper mantle with seismic tomography (Fig. 9). As both composition and temperature can affect seismic wave speeds^58^, it is clear that the Type 6 models (Fig. 5 and Supplementary Fig. S19) best reproduce the major mantle structures below the Tibetan region (Fig. 9). In the preferred model (Run 23, Fig. 5), while prominent cold downwelling developed below subducted Indian lithosphere, as also revealed by seismic tomography (Fig. 9i), most of the underthrusting Indian plate remains coupled with the overriding plate until the present-day (Fig. 5h). According to this model, the shallow-to-flat Indian subduction is important to explain the post-10 Ma shutdown of magmatism in the central Himalayas-Lhasa region (Fig. 9b and Supplementary Fig. S22)^4,34,38^. In other models where the Indian plate maintains normal subduction after ~10 Ma, melting remains active along the trench (Figs. 4o, 9b, 9d, and Supplementary Fig. S6d), i.e., in regions close to the eastern Himalayan Syntaxis (Supplementary Fig. S22), inconsistent with observations in our research area (Fig. 2).

As noted above, we quantitatively evaluated the evolution and crustal responses of different conceptual models (Fig. 1b) using data from the Tibetan Plateau (Fig. 2). By explicitly simulating the melting behaviors (Figs. 5, 7, and 10) and tracking the evolution of the paleo-elevation and crustal thickness (Fig. 9), we have found that the geological history of the Tibetan Plateau, in particular its on-plateau magmatic episodes, helps to further distinguish different model scenarios. Based on this suite of observations (Fig. 2), we propose that the Type 6 model best represents the post-Paleocene subduction history below the Tibetan Plateau. This scenario implies that the Cenozoic subduction process north of India involved three main stages: (1) terrane underthrusting, (2) continental margin subduction, and (3) Indian Subcontinent indentation. However, this model may not be applicable for regions close to the Himalayan syntaxis, where on-plateau geological records differ from those in Fig. 2, and where 3D effects become most significant.

### New constraints on Cenozoic subduction between India and Tibet

This study highlights a potential paleogeographic environment between India and Tibet that potentially contained a ~1400 km long thinned continental margin with a ~600 km long external buoyant continental terrane on the north, a new tectonic configuration that we call “Himalandia”. We emphasize that the morphology of Himalandia alone is insufficient to form the Tibetan Plateau, which would also require other factors, such as weaknesses within the upper plate^4,24,39^. In this scenario, the thinned continental margin could have been mainly covered by seawater, which could also explain the relatively late closure of the “Tethyan Seaway” that happened >20 Myr after the initial collision^4,33,59^. Tectonically, our proposed scenario represents a potential compromise between two earlier concepts of Greater India^17^ and Greater Indian Basin^1^. Its apparent middle ground allows this new model to satisfy the main arguments for both of these endmember hypotheses: the extensive continuous continental subduction in the former and the limited Himalayan crustal mass in the latter^23^. Therefore, this new tectonic scenario appears to better explain the Cenozoic subduction history of South Asia, and can be further tested by future geological and geophysical observations.

Our preferred (Type 6) model predicts that during the collision, a buoyant continental terrane with its upper crust accreted to the overriding plate, while the rest of the lithosphere underthrust to form a flat slab. This new insight can also reconcile several apparent ‘conflicts’ between available observational constraints on Tibetan evolution. For example, while structural geology and the lack of ophiolites along the Yalu Suture zone favor a continuous history of continental underthrusting prior to Indian indentation^18,19,39,60^, paleomagnetic and sedimentary data imply the existence of a broad marine basin around this time, potentially underlain by oceanic crust(s)^1,2,11,59^. Our study suggests that this marine basin could instead be floored by thinned continental crust. Additional numerical models with different slab density structures further confirm that the southern portion of the ‘lost’ lithosphere should be denser than cratonic-like terrane lithosphere but more buoyant than typical oceanic lithosphere (Supplementary Figs. S5–S20, Supplementary Table S2). This could imply either a thinned continental margin, as we suggest here, or a young and buoyant oceanic lithosphere^1,2,11,20^. According to our preferred model, subduction of this lithosphere generated a ~100–200 km wide accretionary wedge by 25 Ma (Fig. 5d), so that some wedge material should have been preserved at the surface till the present (Fig. 5e, f). Consequently, the observed absence of ophiolites within the preserved Himalayan sequences favors the predominance of continental crust covering this lost plate^4^. Although the absence of evidence cannot be undisputable evidence of absence, additional recent geological studies^18,19,39^ and our modeling results further support the idea that the lost lithosphere directly north of the Indian Subcontinent was most likely a thinned continental margin that contained little to no oceanic crust - Himalandia.

## Methods

### Numerical method

We use a MATLAB-based package to conduct the 2-D numerical experiments^56,61–64^. This package is based on the Lagrangian-type finite element code MILAMIN^65^. It has a free surface on the top^66^ and has been benchmarked with a free subduction study^56,62^. A triangular mesh is adaptively generated/regenerated according to the distribution of materials on tracers^56,62,64^.

We assume incompressible viscoplastic rheology (Supplementary Fig. S1) governed by the conservation of mass, moment, and energy (Eqs. (S1)–(S3)). The energy equation (Eq. (S3)) considers diffusion, internal heating, and shear heating (viscous dissipation).

S1 $$\nabla \cdot \mathop{u}\limits^{ \rightharpoonup }=0$$

S2 $$-\nabla p+\nabla \cdot \left[{\eta }_{{eff}}\left(\nabla \mathop{u}\limits^{ \rightharpoonup }+{\nabla }^{T}\mathop{u}\limits^{ \rightharpoonup }\right)\right]+\varDelta \rho \mathop{g}\limits^{ \rightharpoonup }=0$$

S3 $$\rho {c}_{p}\frac{T}{t}=\nabla \cdot \left(k\nabla T\right)+H$$

In Eqs. (S1)–(S3), $\mathop{u}\limits^{ \rightharpoonup }$ is velocity, *p* is dynamic pressure, $\mathop{g}\limits^{ \rightharpoonup }$ is gravity acceleration. The effective viscosity *η*_*eff*_ is calculated with Eqs. (S5) and (S7), considering contributions from diffusion and dislocation creep (Eq. (S4)). *ρ* is density (Eq. (S8)), *c*_*p*_ is heat capacity, *t* is time, *k* is thermal conductivity, and *H* is the volumetric heat production rate (including radioactive heating and viscous dissipation).

S4 $$\eta=\frac{1}{2}{A}^{-\frac{1}{n}}{({\dot{\varepsilon }}_{{II}}^{{\prime} })}^{\frac{1}{n}-1}\exp \left(\frac{(E+p\cdot V)}{{nRT}}\right)$$

S5 $${\eta }_{{eff}}={\left({{\eta }_{{dis}}}^{-1}+{{\eta }_{{dif}}}^{-1}\right)}^{-1}$$

${\dot{\varepsilon }}_{{II}}^{{\prime} }$ is the second invariant of deviatoric strain rate tensor (i.e., Supplementary Fig. S2), *A* is a pre-exponential constant, *E* is the activation energy, *n* describes the exponential dependence of viscosity on ${\dot{\varepsilon }}_{{II}}^{{\prime} }$, *R* is the universal gas constant, and *V* is activation volume. *η*_*dis*_ is viscosity due to dislocation creep, *η*_*dif*_ is viscosity due to diffusion creep.

We use a Drucker–Prager yield criterion to define the visco-plastic transition. When the second invariant of the deviatoric stress tensor (i.e., Supplementary Fig. S3) is larger than the yielding plastic potential Γ (Eq. (S6)), the effective viscosity on the yielding point is calculated with Eq. (S7).

S6 $$\varGamma=p\sin (\varphi )+{c}_{0}\cos (\varphi )$$

where *c*_*0*_ is yielding cohesion, and *φ* is friction angle.

S7 $${\mu }_{{eff}}=\frac{\varGamma }{2{\dot{\varepsilon }}_{{II}}^{{\prime} }}$$

The state equation for density (i.e., Supplementary Fig. S4) is:

S8 $$\rho=({\rho }_{0}+{\varGamma }_{{eclo}} \varDelta {\rho }_{{eclo}}+{\varGamma }_{m} \varDelta {\rho }_{m}) \cdot \exp \left[-{\int }_{{T}_{0}}^{T} \alpha \left(p={p}_{0},T\right){dT}+{\int }_{{p}_{0}}^{p} \frac{{dp}}{K}\right]$$

S9 $$\alpha={\alpha }_{0}+{\alpha }_{1}T+{\alpha }_{2}{T}^{-2}$$

where *ρ*_*0*_ is the reference density, *α* is the temperature-dependent thermal expansion coefficient, *α*_*0*_ is *α* at T_0_ = 20 °C, *p*_*0*_ is atmospheric pressure, *K* is the bulk modulus (assumed to be constant), and *T* and *p* are the temperature (in Kelvin) and pressure, ${\varGamma }_{{eclo}}$ is defined in Eq. (S10) (for the upper crust and mantle materials, this parameter is 0), $\varDelta {\rho }_{{eclo}}$ is density contrast after the complete eclogitization (3.37 g/cm^3^-${\rho }_{0}$), ${\varGamma }_{m}$ (for crustal materials, this parameter is 0) and $\varDelta {\rho }_{m}$ are defined in Eq. (S12).

The eclogite phase transformation of mafic crustal materials (oceanic crust, the lower crust of overriding plate, and the ICP lower crust) is simulated using Eqs. (S10) and (S11)^67^. After the complete eclogitization, the compositional density of crustal materials increases to 3.37 g/cm^3^.

S10 $${\varGamma }_{{eclo}}=1-\exp \left(-{\left({A}_{{kin}}{Yt}\right)}^{4}\right)$$

where *Γ*_*eclo*_ is the duration of the eclogitization reaction, *t* is time, and *A*_*kin*_ determines the temperature dependence of eclogitization.

S11 $$Y\sim T\exp \left(\frac{-{E}_{{kin}}^{*}}{{RT}}\right)\left[1-\exp (\frac{\varDelta G}{{RT}})\right]$$

*Y* is the Arrhenius formulation of the growth function^67,68^, where *T* is temperature, ${E}_{kin}^{\ast }$ is the activation energy for growth (214 kJ), *R* is the gas constant, and *ΔG* is the Gibbs free energy difference between basalt and eclogite. Parameters for calculating the eclogitization history are from van Hunen et al., 2002, 2004.

Phase changes of mantle materials (olivine to wadsleyite and ringwoodite to bridgmanite) are calculated with Eq. S12^69,70^.

S12 $${\varGamma }_{m}=\frac{1}{2}\left(1+\tanh \left[\frac{z-{z}_{t}-{\gamma }_{c}\left(T-{T}_{t}\right)}{w}\right]\right)$$

where *Γ*_*m*_ is the duration of phase change reaction, *z* is depth, *z*_*t*_ and *T*_*t*_ are respectively the average depth and temperature of the phase transformation, *w* is the half-width of the phase transformation, ${\gamma }_{c}$ is the Clapeyron slope. Parameters in the olivine to wadsleyite transformation: ${\gamma }_{c}$= 3.1 MPa/K, *z*_*t*_ is 410 km, *T*_*t*_ is 1760 K, *w* is 25 km, and density contrast ($\varDelta {\rho }_{m}$) after the complete transformation is 0.28 g/cm^3^. Parameters in the ringwoodite to bridgmanite transformation: ${\gamma }_{c}$= -2.8 MPa/K, *z*_*t*_ is 660 km, *T*_*t*_ is 1870 K, *w* is 25 km, and density contrast ($\varDelta {\rho }_{m}$) after the complete transformation is 0.342 g/cm^3^.

We calculate the melting locations based on the method in Liu et al., 2018a, 2018b. In addition, we have considered the effects of melting degree *f* and source mineral assemblages (asthenospheric vs. lithospheric mantle) on the solidus^56,62,71,72^ (Fig. 10). For each time step, the melting process is modeled using the following procedures:

1. Heat-induced melting. If temperature *T*_*a*_ (transformed from the potential temperature *T* with a 0.3 K/km adiabatic gradient) lies above the solidus *T*_*m*_ (Eq. S13), f will increase by a heat-induced melting degree increment $d{f}_{h{eat}}$ (Eq. S14). Thus, the temperature at melting locations is updated to the new solidus (Eq. S13).

S13 $${T}_{m}={T}_{0}^{m}+{\left(\frac{\partial {T}^{m}}{\partial p}\right)}_{f}p+{\left(\frac{\partial {T}^{m}}{\partial f}\right)}_{p}f$$

S14 $${\left(\frac{{df}}{{dT}}\right)}_{{heat}}=\frac{1}{\left[{\left(\frac{\partial {T}^{m}}{\partial f}\right)}_{p}+\frac{{Q}_{L}}{{c}_{p}}\right]}$$

where ${T}_{0}^{m}$ is the solidus at room temperature and pressure, *Q*_*L*_ is latent heat during melting, ${(\frac{\partial {T}^{m}}{\partial p})}_{f}$ describes the dependence of solidus on *p*, ${(\frac{\partial {T}^{m}}{\partial f})}_{p}$ describes the dependence of solidus on *f*, *c*_*p*_ is heat capacity (Supplementary Table S1). For the mantle materials^71^: ${T}_{0}^{m}$is 1081 and 1136 °C for the asthenospheric and lithospheric mantle, respectively; ${(\frac{\partial {T}^{m}}{\partial p})}_{f}$ is 132 K/GPa; ${(\frac{\partial {T}^{m}}{\partial f})}_{p}$ is 250 K, *Q*_*L*_ is 400 kJ/kg. For the crustal materials: ${T}_{0}^{m}$ is based on the results of lab experiments^73^; ${(\frac{\partial {T}^{m}}{\partial f})}_{p}$ and ${(\frac{\partial {T}^{m}}{\partial p})}_{f}$ are linearly interpolated according to the solidi in Fig. 10, based on *T*, *p*, and *f*; *Q*_*L*_ is 380 kJ/kg^71^.

2. Decompression melting. During decompression upwelling, a pressure decrease *dp* may allow materials to reach their solidi at new depths. In this scenario, *f* and new solidus are updated (Eq. S13). The melting degree increment *df* due to decompression is modeled with Eq. S15. The temperature at melting locations is then updated to the new solidus (Eq. S13).

S15 $$-{\left(\frac{{df}}{{dp}}\right)}_{s}=\frac{\left[{\left(\frac{\partial {T}^{m}}{\partial p}\right)}_{f}-\frac{\left(\alpha T\right)}{\left(\rho {c}_{p}\right)}\right]}{\left[\frac{{Q}_{L}}{{c}_{p}+{\left(\frac{\partial {T}^{m}}{\partial f}\right)}_{p}}\right]}$$

where $\alpha$ is thermal expansion (Eq. (S9)).

For increasing numerical stability, the melting increment is calculated iteratively when *df*_*heat*_ > *δf* (i.e., *δf* = 0.01) in Eq. (S14) or dp > *δp* (i.e., *δp* = 0.001 GPa) in Eq. (S15).

The locations of oceanic slab dehydration (Fig. 5) are interpolated from the slab-mode databases in Kimura (2017), based on *p* and *T* on tracers. Specifically, the databases of MORB and Depleted MORB Source^74^ are used for oceanic crust and lithospheric mantle, respectively. The transient velocity for melting/dehydration tracers is calculated with Equations 13-14 (*cf*. Chen and Faccenda et al., 2019)^75^.

S16 $${v}_{x}={v}_{x0}-\frac{{K}_{p}}{\left(\varPhi \times \eta \right)}\times \frac{\partial p}{\partial x}$$

S17 $${v}_{y}={v}_{y0}-\frac{{K}_{p}}{\left(\varPhi \times \eta \right)}\times \left(\frac{\partial p}{\partial x}-{\rho }_{f}g\right)$$

where ${v}_{x0}$ and ${v}_{y0}$ are respectively the local horizontal and vertical velocity, ${v}_{x}$ and ${v}_{y}$ are respectively the horizontal and vertical velocity of fluid/melt tracers, *g* is gravitational acceleration, ${K}_{p}$ is permeability, $\varPhi$ is porosity, $\eta$ is the local viscosity, and ${\rho }_{f}$ is density (1 g/cm^3^ for water, and 2.6 g/cm^3^ for melts). For simplicity, we followed Chen and Faccenda (2019) and set $\frac{{K}_{p}}{\left(\varPhi \cdot \eta \right)}$ as a constant $1.33\times 1{0}^{-13}{m}^{3}/{kg}$.

### Model setup

The 2D numerical box is 3500 km wide × 1500 km deep (Fig. 3, Supplementary Table S2). In all numerical models, the best resolution is ~5 km, where the mesh node is within 50 km from the nearest material interface, and it gradually increases to ~50 km, where the node is >190 km away from the closest material interface. We added the time-dependent convergence rate (Fig. 1a) for the incoming plate between 10 and 100 km (along the horizontal direction) and above the 400 km depth (cf. Gerya and Meilick, 2011)^76^. The surface and bottom temperatures remain at 0 and 1350 °C, respectively. All continental plates initially follow a 1-D steady-state conductive thermal profile. The oceanic slab tip is 80 Myr old, similar to the Tethyan Ocean during the early Cenozoic^11,12^. The overriding plate composes (1) a 1300 km long weak portion (with the yielding stress capped at 50 MPa) on the trench side and (2) an 1100 km long strong portion (with the yielding stress capped at 200 MPa) to the far side. The weak and strong portions mimic the Tibetan terranes (Lhasa, Qiangtang, Hoh-Xil) and the Asian continent (i.e., Eastern Kunlun-Qaidam), respectively^43,44,77^. In addition, the length of the weak portion approximates that of Tibetan terranes before the collision in the early Eocene^4,39^, and its lithospheric mantle could delaminate during the continental collision^77^.

The incoming plate before the Indian Subcontinent is purely continental in the Type 1 models, including a ~2000 km long “Greater India”, while that in the Type 2 models is purely oceanic (Fig. 1b, Fig. 3a vs. 3b). The Types 3 and 4 models consider a moderately long (900 km) and short (~200 km) Greater India, respectively, following a respective ~1100 and 1800 km long oceanic plate^4,21^. In the Types 5 models, the incoming plates north of the Indian Subcontinent compose a 500–600 km long continental portion (with a compositional density of 3.37 g/cm^3^) and a ~1500–1400 km long oceanic portion (Fig. 3c, Supplementary Table S2). In the Type 6 models, the incoming plates north of the Indian Subcontinent compose a 600 km long continental portion and a ~1400 km long thinned-margin-like portion (Fig. 3d, Supplementary Table S2). In addition, we presume a mafic crust and ~140 km thick lithospheric mantle for the Indian plate (Supplementary Table S1) (*cf*., Singh et al., 2017)^78^.

All models simulate a 55 Myr evolution, and the total convergence is ~3150 km (Fig. 1a). We also explicitly calculate the melting history in all models (Fig. 10). Further details of the model setup and parameters are discussed in the supplementary material.

## Supplementary information

Supplementary information

Description of Additional Supplementary files

Supplementary Movie 1

Supplementary Movie 2

Supplementary Movie 3

Supplementary Movie 4

Supplementary Movie 5

Supplementary Movie 6

Supplementary Movie 7

Supplementary Movie 8

## Data Availability

The datasets used in compiling Fig. 2 are available from the cited papers. Modeling results are calculated from the equations in the methods section and are presented in the main and supplementary figures and supplementary movies.

## Code availability

The computer code used for this paper and parameter choices for the model runs shown here are available at Zenodo (10.5281/zenodo.6836907) and supplements.
