# Supplementary material for: New constraints on Cenozoic subduction between India and Tibet
Source: Nat Commun. 2023 Apr 7;14:1963. doi: 10.1038/s41467-023-37615-5 (PMC10082029; doi:10.1038/s41467-023-37615-5)
Supplement: Supplementary file 1 — Supplementary information [file 41467_2023_37615_MOESM1_ESM.pdf]

New Constraints on Cenozoic Subduction Between India and Tibet

Liang Liu[1,2,3]*, Lijun Liu[2**], Jason. P. Morgan[4], Yi-Gang Xu[1,3], and Ling Chen[5]

1. State Key Laboratory of Isotope Geochemistry and CAS center of Excellence in Deep Earth Science, Guangzhou Institute of Geochemistry, Chinese Academy of Science, Guangzhou, 510640, China.

2. Department of Geology, University of Illinois at Urbana-Champaign, Urbana, 61801, USA.

3. Southern Marine Science and Engineering Guangdong Laboratory (Guangzhou), Guangzhou, 511458, China.

4. Department of Marine Science and Engineering, Southern University of Science and Technology, Shenzhen, Guangdong 518055, China.

5. State Key Laboratory of Lithospheric Evolution, Institute of Geology and Geophysics, Chinese Academy of Sciences, Beijing, China

* Liang Liu: llsinogm@yeah.net

** Lijun Liu: ljliu@illinois.edu

This file provides additional supporting information to the main text. It contains the result summaries of further sensitivity tests, two tables, and twenty-two figures.

1. Supplementary Notes

As mentioned in the main text, we tested different compositional densities for the incoming plates in the numerical models (Figures S5, S7, S9, S11, S19). The density of the incoming continental lithospheric mantle (ICLM, including the cratonic Indian plate) is found to not obviously affect the final plateau width but can determine the final plateau elevation. In these sensitivity tests, the melting patterns change slightly when the ICLM compositional density is ≤3.36 g/cm^3 (Figures S6, S8, S10, S12). In the scenarios where the cratonic Indian plate develops steep subduction, the melting close to the suture keeps active at the end of the model (Figures S6d, S16d, S19), in contrast to the observations (Figures 2a-2b).

In the **Type 6** models, except for the effects of the ICLM, we also tested the effects of the overriding-plate viscosity (Figure S17), terrane slab rheology (Figure S18), and lower mantle viscosity (Figure S20):

1) The maximum yielding stress of the overriding-plate crust does not noticeably affect the terrane slab evolution (Figures S17a-S17c); however, if the lithospheric mantle of the overriding plate strongly resists shortening, the initiation of the flat terrane subduction is delayed by ~9 Myr, leading to a distinctive evolution of the lithospheric deformation and melting (Figure S17d).

2) We found that the rapid roll-back of the oceanic slab can drag the terrane towards the trench (Figures 5e-5f)[1,2]. This process tears the terrane slab into two parts, while the scale of the relic terrane beneath the overriding plate highly depends on the rheology of the terrane slab (Figures 5, S18). An extensive relic terrane can protect the post-delamination Tibetan lithosphere from the hot underlying mantle (Figure 5d, 7f) and thus, may help explain the magmatic hiatus in the central plateau after ~25 Ma (Figure 2b). The low yield stress (20 MPa) that we assume for the terrane slab during the roll-back of the oceanic slab (Figure 5) is mostly believed to be due to the fluids released from the underlying slab (Figure 5e) (*cf.*, Hirth and Kohlstedt, 2003)[3]. As we did not explicitly calculate the effect of fluids on yielding behavior, this parameter choice is largely suppositional but within the estimated ranges for lithospheric deformation (*cf.*, Ghosh and Holt, 2012)[4]. However, this sensitivity test does not improve on the melting prediction of Model 5 to better match observations, since its dense oceanic slab detaches from the surface plate as early as ~40 Ma, leading to continuous melting in the southern plateau until Indian underthrusting, inconsistent with observations (Figure 7).

3) The lower mantle viscosity controls the sinking rate of slabs (Figure S20). The slab sinking rate remains unchanged when the lower mantle viscosity is larger than 5×10^{22} Pa·s (Figures S20d-S20f), and in these scenarios, former slabs can partially remain within the upper mantle (Figures S20).

Further sensitivity tests regarding the length, rheology, and density of incoming plates, the rheology of the overriding plates, and the convergence rate are also in Liu et al., 2021a[1].

2. Supplementary Tables

Materials	1	2	3	4	5	6	7	8	9	10	11	12
Flow Law Parameters (Ref. 1-8)												
A_{dis} ($Pa^{-n}\cdot s^{-1}$)	$10^{-15.05}$	$4.85\cdot10^{-17}$	$4.85\cdot10^{-17}$	$4.85\cdot10^{-17}$	$4.85\cdot10^{-17}$	$3.3\cdot10^{-21.2}$	$1.8\cdot10^{-32}$	$6.7\cdot10^{-20.4}$	$10^{-5.3}$	$10^{-5.3}$	$10^{-7.3}$	$1.8\cdot10^{-32}$
n_{dis}	3.5	3.5	3.5	3.5	3.5	3.2	4.0	2.4	3	3	3	4.0
E_{dis} (kJ/mol)	480	535	535	535	535	238	137	156	648	648	648	137
V_{dis} (cm^3/mol)	8	8	8	8	8	1	8	8	1	8	1	8
A_{dif} ($Pa^{-n}\cdot s^{-1}$)	$10^{-8.65}$	$10^{-8.65}$	$10^{-8.65}$	$10^{-8.65}$	$10^{-8.65}$	$4.0\cdot10^{-8}$	$4.0\cdot10^{-7}$	$4.0\cdot10^{-13}$	$10^{6.1}$	$10^{6.1}$	$10^{4.1}$	$4.0\cdot10^{-7}$
n_{dif}	1	1	1	1	1	1	1	1	1	1	1	1
E_{dif} (kJ/mol)	335	375	375	375	375	153	220	220	476	476	476	220
V_{dif} (cm^3/mol)	4	4	4	4	4	1	4	4	1	4	1	4
c_0 (Mpa)	20	20	20	20	2	20	2	20	2	20	2	2
φ (º)	ICP middle crust, and WOP materials: 30 (γ = 0) → 2 (γ > 1); other materials: 30 (γ = 0) → 15 (γ > 1)											
Thermal Parameters (Ref. 2)												
k ($Wm^{-1}K^{-1}$)	3.3	3.3	3.3	3.3	3.3	2.5	2.5	2.5	2.5	2.5	2.5	2.5
H_r (µW/m^3)	0.03	0.03	0.03	0.03	0.03	0.25	1.5	1.5	1	0.25	0.25	1.5
c_p (J/(kg·K)	1200	1200	1200	1200	1200	1200	1200	1200	1200	1200	1200	1200
Density Parameters (Ref. 9-10)												

ρ_0 (g·cm^{-3})	3.39	Table S2	Table S2	3.34	3.34	3.00	2.70	2.70	2.80	2.85	2.90	2.90
$a_1(10^{-4})$	0.27768	0.27014	0.27768	0.27014	0.27768	0.27768	0.27014	0.27014	0.27014	0.27014	0.27014	0.27014
$a_2(10^{-8}$ K$^{-1})$	0.95451	1.05945	0.95451	1.05945	0.95451	0.95451	1.05945	1.05945	1.05945	1.05945	1.05945	1.05945
a_3 (K^2)	-0.12404	-0.1243	-0.12404	-0.1243	-0.12404	-0.12404	-0.1243	-0.1243	-0.1243	-0.1243	-0.1243	-0.1243
K (Gpa^{-1})	128	130	128	130	128	63	63	63	63	63	63	63

Table S1. Parameters in numerical experiments. Materials: 1- Asthenosphere (Ref. 1-2), 2- ICP lithospheric mantle (Ref. 1-2), 3- IOP lithospheric mantle (Ref. 1-2), 4- SOP lithospheric mantle, 5-WOP lithospheric mantle (Ref. 1-2), 6- IOP crust (Ref. 4-7), 7- ICP upper crust (Ref. 3-4), 8- Upper crust of overriding plates (Ref. 4-7), 9- ICP middle crust (Ref. 4-7), 10- Continental lower crust (including the lower crust of the ICP, overriding plates, and Indian lower crust) (Ref. 8), 11- Indian middle crust (Ref. 3-4), 12- Indian upper crust (Ref. 4-7). ICP-Incoming Continental Plate (i.e., Greater India, continental terranes, or the continental margin in the **Type 6** models). IOP-Incoming Oceanic Plate (i.e., Greater Indian Basin or back-arc basin). SOP-Strong Overriding Plate (i.e., Asian interiors). WOP-Weak Overriding Plate (i.e., Tibetan terranes).

The parameters for **oceanic sediments** are the same as those of Material 8, except for ρ_0 = 2.60 g/cm^3. The parameters for the **continental margin crust** are the same as those of Material 6. The parameters for the **continental margin lithospheric mantle** are the same as those of Material 2, except for ρ_0 = 3.37 g/cm^3. The parameters for the **"Indian"** lower crust and lithospheric mantle are the same as those of the ICP, except that ρ_0 (Indian lower crust) = 3.0 g/cm^3, and A mafic feature of the Indian crust is implied in the results of seismic tomography[5]. In Runs 32-35, ρ_0 (Indian lithospheric mantle) is 3.35 g/cm^3, 3.36 g/cm^3, 3.37 g/cm^3, and 3.38 g/cm^3, respectively; in other models, ρ_0 (Indian lithospheric mantle) is 3.34 g/cm^3. Except for those be highlighted in Table S2: the maximum yielding stress for the ICP crust and mantle is 100 MPa, that for WOP materials is 50 MPa, and that for other materials is 200 MPa.

Subscript "dis" refers to parameters for dislocation creep, while "dif" refers to diffusion creep (Equation S5). For the depth <660 km, the material viscosity is between 10^{19} Pa·s and 10^{24} Pa·s; For greater depths, we assume that the diffusion creep is the only deformation regime in the mantle, whose viscosity is confined to be $\leq 10^{23}$ Pa·s (Ref. 11-12). c_0- yielding cohesion, φ- friction angle, γ is the accumulated plastic strain. k- thermal conductivity, H_r- radioactive heat production, c_p- heat capacity, ρ_0- the reference density at room temperature (20℃) and pressure (0.1 MPa), K- bulk modulus.

References. 1- Hirth and Kohlstedt, 2003[3]; 2- Brune et al., 2014[6]; 3- Gleason and Tullis, 1995[7]; 4- Rutter and Brodie, 2004[8]; 5- Kirby and Kronenberg, 1987[9]; 6- Ranalli and Murphy, 1987[10]; 7- Huangfu et al., 2018[11]; 8- Rybacki and Dresen, 2004[12]; 9- Djomani et al., 2001[13]; 10- Schutt and Lesher, 2006[14]; 11- Karato and Wu, 1993[15]; 12- Yamazaki and Karato, 2001[16]

Run No.	Mode l	M.Y.S. (W.O. C.)	M.Y.S. (W.O.L.M.)	M.Y.S. (terrane hinge)	ICP mantle C. Density	IOP mantle C. Density	Lower Mantle Viscosity	Revelant Figures and Movies
1	Type 1	50 MPa	50 MPa	N/A	3.32 g/cm^3	N/A	3×10^{22} Pa·s	S5, S6
2	Type 1	50 MPa	50 MPa	N/A	3.30 g/cm^3	N/A	3×10^{22} Pa·s	S5, S6
3	Type 1	50 MPa	50 MPa	N/A	3.36 g/cm^3	N/A	3×10^{22} Pa·s	6-8, S5,S6
4	Type 1	50 MPa	50 MPa	N/A	3.38 g/cm^3	N/A	3×10^{22} Pa·s	5, S5, S6, Movie 1
5	Type 2	50 MPa	50 MPa	N/A	N/A	3.37 g/cm^3	3×10^{22} Pa·s	S7, S8
6	Type 2	50 MPa	50 MPa	N/A	N/A	3.39 g/cm^3	3×10^{22} Pa·s	5-8, S7, S8, Movie 2
7	Type 3	50 MPa	50 MPa	N/A	3.32 g/cm^3	3.37 g/cm^3	3×10^{22} Pa·s	S9, S10
8	Type 3	50 MPa	50 MPa	N/A	3.32 g/cm^3	3.37 g/cm^3	3×10^{22} Pa·s	S9, S10
9	Type 3	50 MPa	50 MPa	N/A	3.36 g/cm^3	3.37 g/cm^3	3×10^{22} Pa·s	5-8, S9, S10,Movie 3
10	Type 3	50 MPa	50 MPa	N/A	3.38 g/cm^3	3.37 g/cm^3	3×10^{22} Pa·s	S9, S10, Movie 3
11	Type 4	50 MPa	50 MPa	N/A	3.32 g/cm^3	3.37 g/cm^3	3×10^{22} Pa·s	S11, S12
12	Type 4	50 MPa	50 MPa	N/A	3.34 g/cm^3	3.37 g/cm^3	3×10^{22} Pa·s	S11, S12
13	Type 4	50 MPa	50 MPa	N/A	3.36 g/cm^3	3.37 g/cm^3	3×10^{22} Pa·s	5-8, S11, S12,Movie 4
14	Type 4	50 MPa	50 MPa	N/A	3380 g/cm^3	3.37 g/cm^3	3×10^{22} Pa·s	S11, S12
15	Type 5	50 MPa	50 MPa	N/A	See caption	3.39 g/cm^3	3×10^{22} Pa·s	S13, S14, Movie 5
16	Type 5	50 MPa	100 MPa	N/A	See caption	3.39 g/cm^3	3×10^{22} Pa·s	S13, S14
17	Type 5	50 MPa	150 MPa	N/A	See caption	3.39 g/cm^3	3×10^{22} Pa·s	S13, S14
18	Type 5	50 MPa	200 MPa	N/A	See caption	3.39 g/cm^3	3×10^{22} Pa·s	5-8, S13,S14, Movie 6
19	Type 5	50 MPa	50 MPa	N/A	See caption	3.39 g/cm^3	3×10^{22} Pa·s	S15, S16
20	Type 5	50 MPa	100 MPa	N/A	See caption	3.39 g/cm^3	3×10^{22} Pa·s	S15, S16

21	Type 5	50 MPa	150 MPa	N/A	See caption	3.39 g/cm^3	3×10^{22} Pa·s	S15, S16
22	Type 5	50 MPa	200 MPa	N/A	See caption	3.39 g/cm^3	3×10^{22} Pa·s	S15,S16
23	Type 6	50 MPa	50 Mpa	20 MPa	See caption	N/A	10×10^{22} Pa·s	4-8, S1-S4,S20, Movies 7-8
24	Type 6	100 MPa	50 MPa	20 MPa	See caption	N/A	3×10^{22} Pa·s	S17
25	Type 6	150 MPa	50 MPa	20 MPa	See caption	N/A	3×10^{22} Pa·s	S17
26	Type 6	200 MPa	50 MPa	20 MPa	See caption	N/A	3×10^{22} Pa·s	S17
27	Type 6	50 MPa	100 MPa	20 MPa	See caption	N/A	3×10^{22} Pa·s	S17
28	Type 6	50 MPa	50 MPa	40 MPa	See caption	N/A	3×10^{22} Pa·s	S18
29	Type 6	50 MPa	50 MPa	60 MPa	See caption	N/A	3×10^{22} Pa·s	S18
30	Type 6	50 MPa	50 MPa	80 MPa	See caption	N/A	3×10^{22} Pa·s	S18
31	Type 6	50 MPa	50 MPa	100 MPa	See caption	N/A	3×10^{22} Pa·s	S18
32	Type 6	50 MPa	50 MPa	20 MPa	See caption	N/A	3×10^{22} Pa·s	S19
33	Type 6	50 MPa	50 MPa	20 MPa	See caption	N/A	3×10^{22} Pa·s	S19
34	Type 6	50 MPa	50 MPa	20 MPa	See caption	N/A	3×10^{22} Pa·s	S19
35	Type 6	50 MPa	50 MPa	20 MPa	See caption	N/A	3×10^{22} Pa·s	S19
36	Type 6	50 MPa	50 MPa	20 Mpa	See caption	N/A	10^{22} Pa·s	S20
37	Type 6	50 MPa	50 MPa	20 Mpa	See caption	N/A	3×10^{22} Pa·s	S20
38	Type 6	50 MPa	50 MPa	20 Mpa	See caption	N/A	5×10^{22} Pa·s	S20
39	Type 6	50 MPa	50 MPa	20 Mpa	See caption	N/A	20×10^{22} Pa·s	S20
40	Type 6	50 MPa	50 MPa	20 Mpa	See caption	N/A	50×10^{22} Pa·s	S20

Table S2. The presented numerical models and their key variables. M.Y.S.- Maximum Yielding Stress. W.O.C.- Weak Overriding-plate Crust (Figure 3). W.O.L.M.- Weak Overriding-plate Lithospheric Mantle (Figure 3). C. Density- Compositional Density (Equation S8). The terrane hinge refers to the portion of the terrane slab above the oceanic slab (Figures 4e, S18), which is assumed to be weakened by dehydration of the oceanic slab (Supplementary Section 1). In the **Type**

5 models, the compositional density of the terrane lithospheric mantle is 3.37 g/cm^3. The terrane Lithosphere is 500 km long in Runs 15-18 and 600 km long in Runs 19-22. In the **Type 6** models: the compositional density of the terrane lithospheric mantle is 3.34 g/cm^3. Other parameters for the numerical models are in Table S1.

3. Supplementary Figures

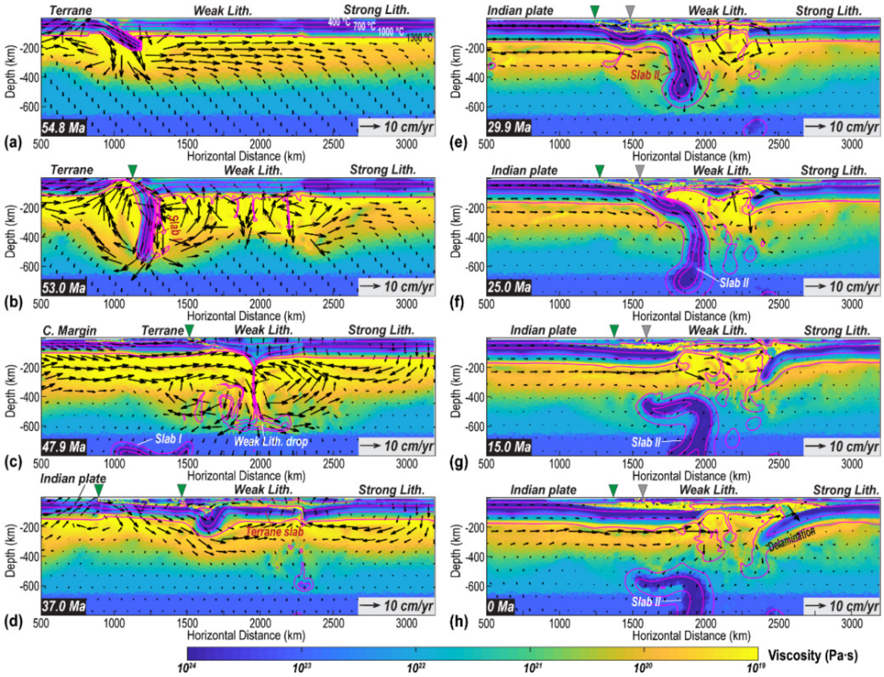

Figure S1. **Evolution of the viscosity filed in the Type 6 model** (Run 23). Lith.- Lithosphere. Slabs I and II demonstrate the same slabs as those in Figure 5. C. Margin- Continental Margin.

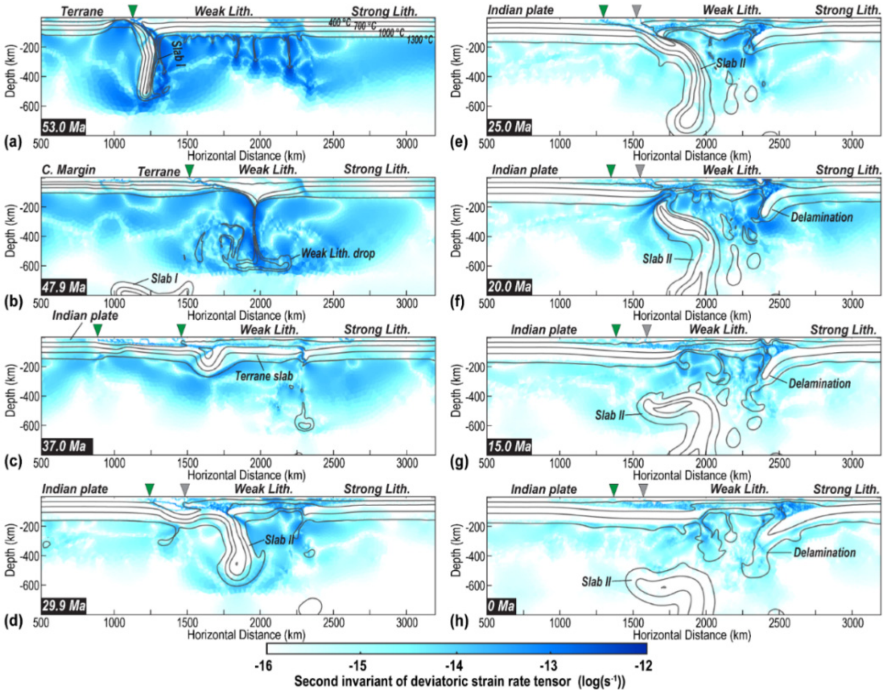

Figure S2. **Evolution of the second invariant of deviatoric strain rate tensor ($\dot{\varepsilon}'_{II}$) in the Type 6 model** (Run 23). Lith.- Lithosphere. Slabs I and II demonstrate the same slabs as those in Figure 5. C. Margin- Continental Margin.

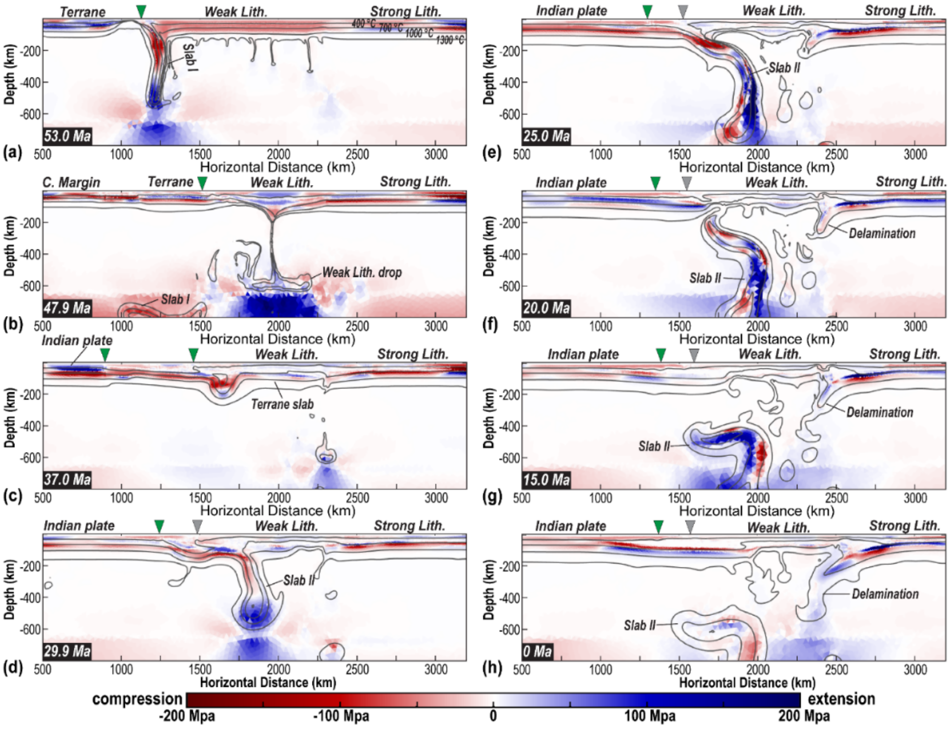

Figure S3. **Evolution of the horizontal deviatoric stress in the Type 6 model** (Run 23). Lith.- Lithosphere. Slabs I and II demonstrate the same slabs as those in Figure 5. C. Margin- Continental Margin.

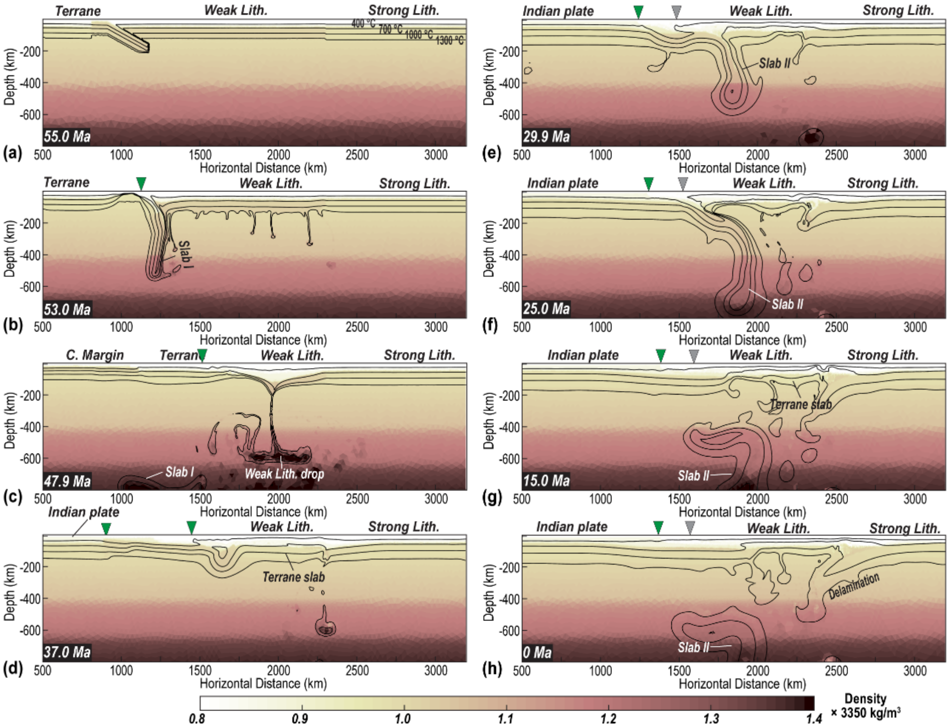

Figure S4. **Evolution of the density field in the Type 6 model** (Run 23). Lith.- Lithosphere. Slabs I and II demonstrate the same slabs as those in Figure 5. C. Margin- Continental Margin.

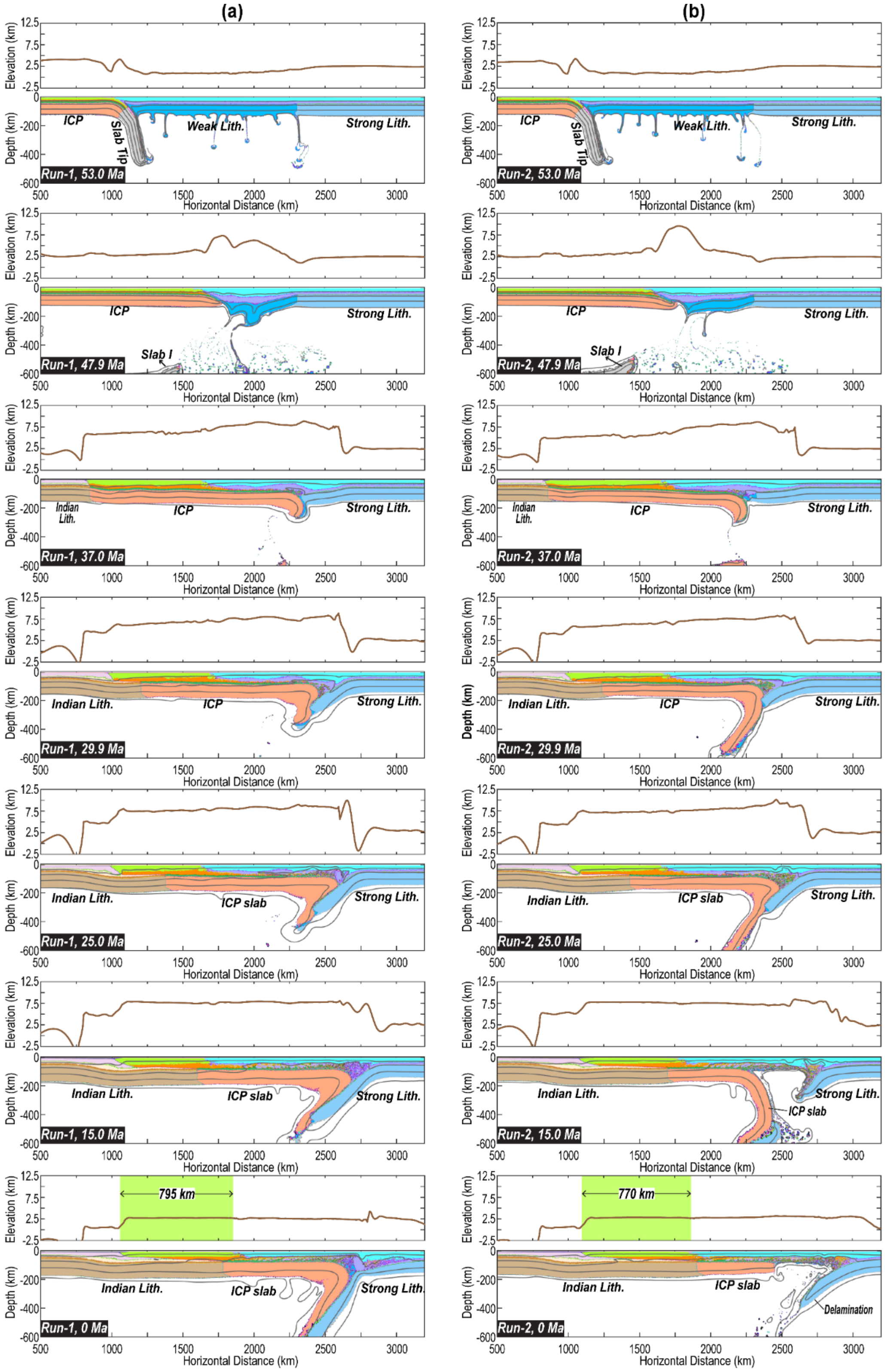

Figure S5. **Evolution of material field and surface topography in the Type 1 models (purely continental subduction)**. (**a**) Results of Run 1. (**b**) Results of Run 2. (**c**) Results of Run 3. (**d**) Results of

Run 4. The green shading with a number in each bottom panel illustrates the width of the accreted crust (*i.e.*, the Tethyan-Greater Himalayas). The compositional density for the ICP lithospheric mantle in each run is: 3.32 g/cm^3, 3.34 g/cm^3, 3.36 g/cm^3, 3.38 g/cm^3, respectively. Other key parameters of these models are in Tables S1-S2. Other plotting habits are the same as those in Figure 5. ICP-Incoming Continental Plate (i.e., Greater India). Lith.- Lithosphere.

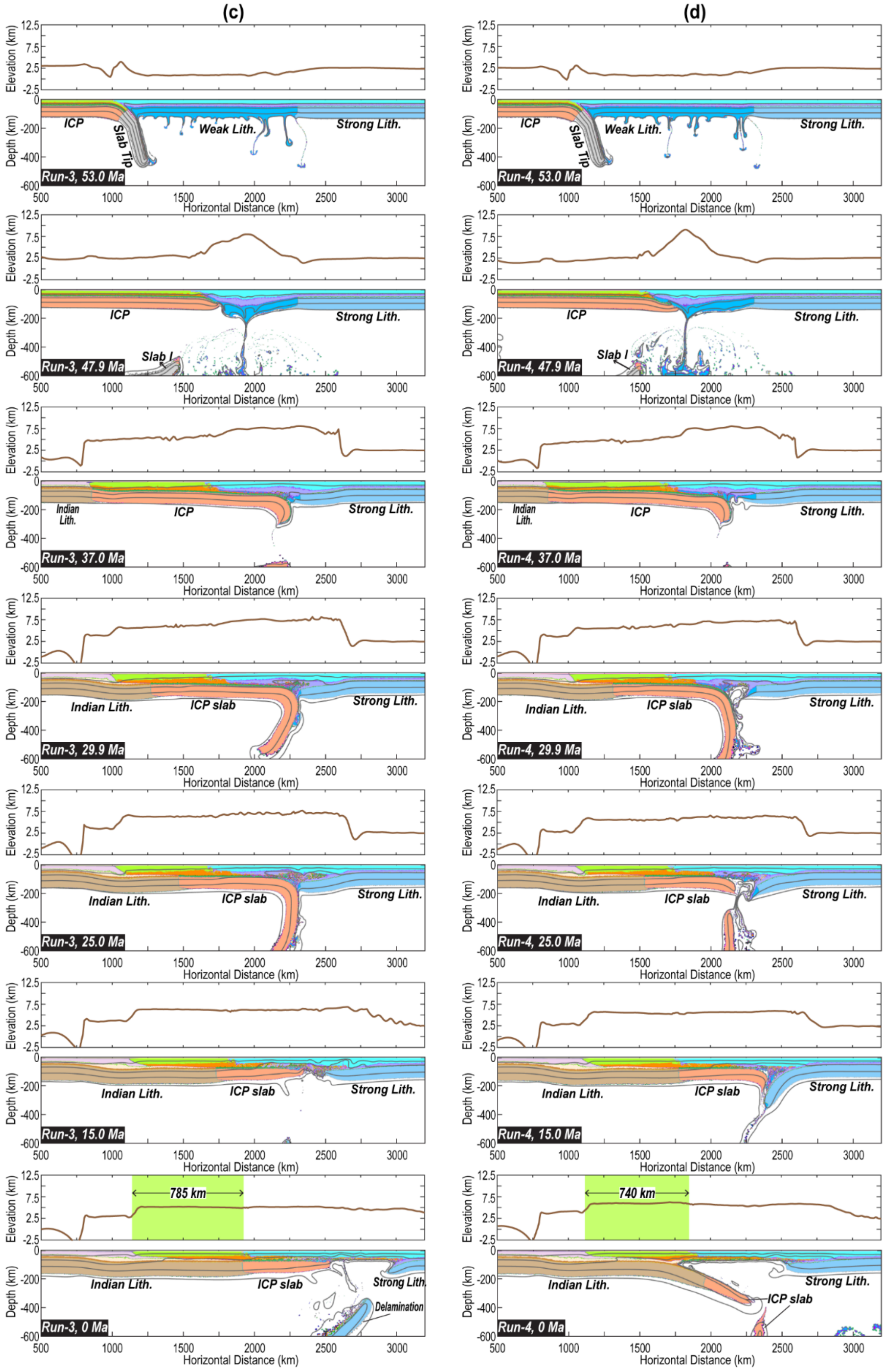

Figure S5 (continued).

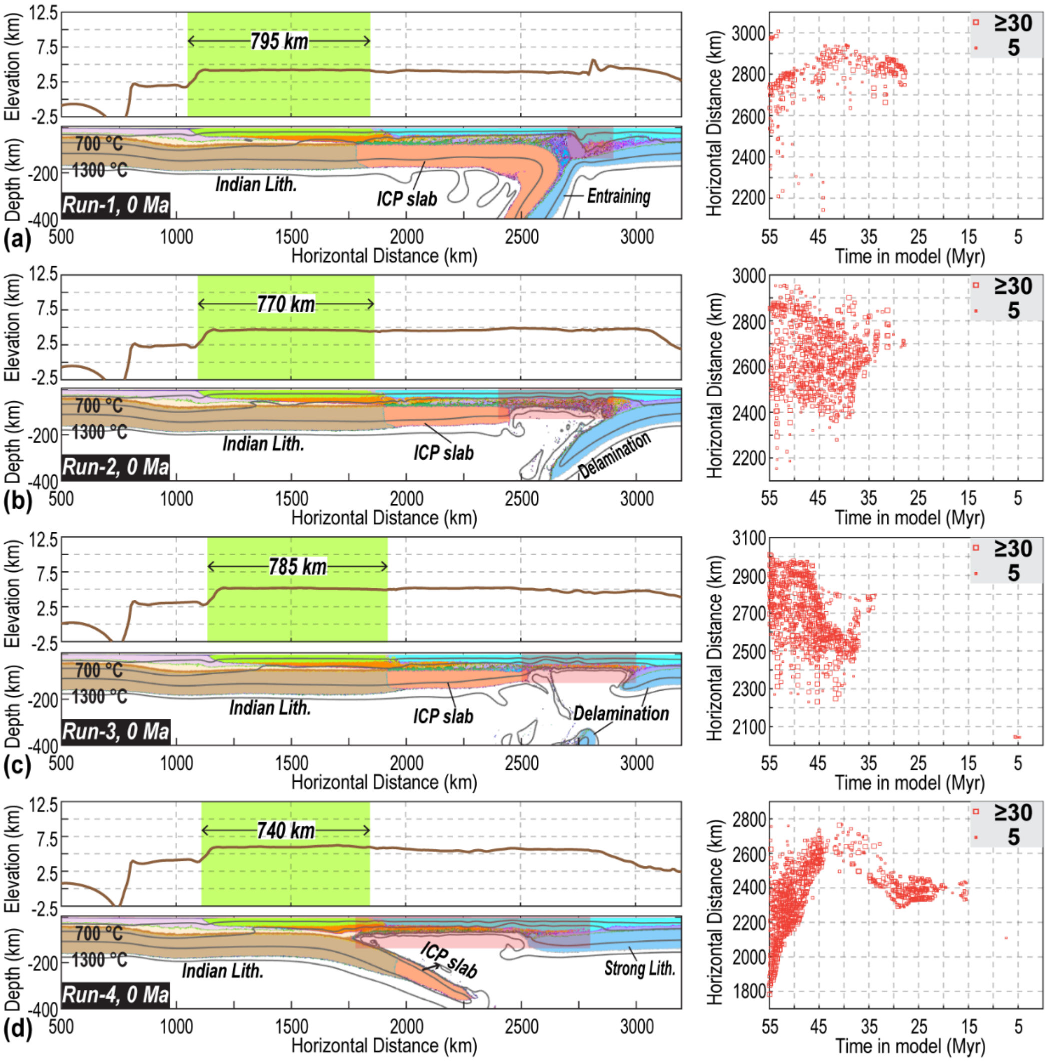

Figure S6. **"Present-day" material field, surface topography, and the melting distribution in the Type 1 models**. (**a**) Results of Run 1. (**b**) Results of Run 2. (**c**) Results of Run 3. (**d**) Results of Run 4. The green shading with a number in each elevation panel illustrates the width of the accreted crust (*i.e.*, Tethyan-Greater Himalayas). The red shading in each material panel highlights the spatial range of melting records. The key parameters of these models are in Tables S1-S2. The plotting habits for melting distribution are the same as those in Figure 7. ICP-Incoming Continental Plate (i.e., Greater India). Lith.- Lithosphere.

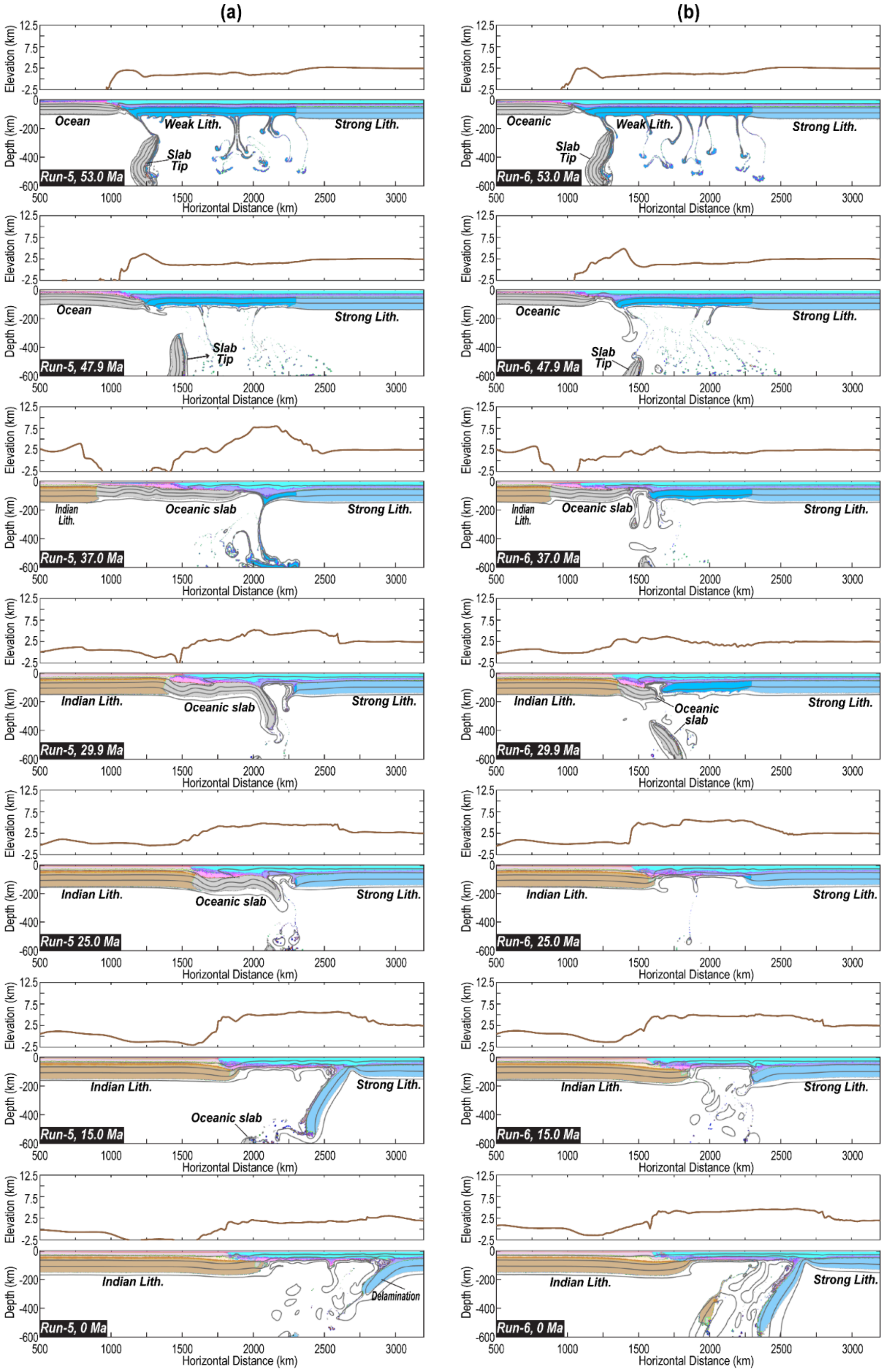

Figure S7. Evolution of material field and surface topography in the Type 2 models (purely oceanic subduction). (**a**) Results of Run 5. (**b**) Results of Run 6. In each run, the compositional density for the

oceanic lithospheric mantle is: 3.37 g/cm^3 and 3.39 g/cm^3, respectively. The oceanic segment is 40 Myr. Other key parameters of these models are in Tables S1-S2. Other plotting habits are the same as those in Figure 5. IOP-Incoming Oceanic Plate (i.e., Greater Indian Basin). Lith.- Lithosphere.

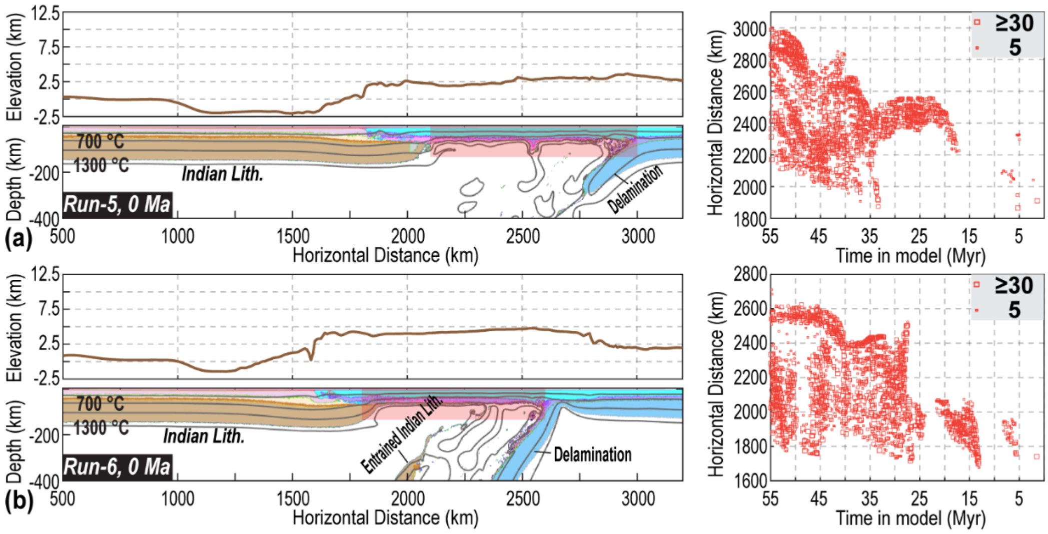

Figure S8. "Present-day" material field, surface topography, and the melting distribution in the Type 2 models. (**a**) Results of Run 5. (**b**) Results of Run 6. The red shading in each material panel highlights the spatial range of melting records. The key parameters of these models are in Tables S1-S2. The plotting habits for melting distribution are the same as those in Figure 7. Lith.- Lithosphere.

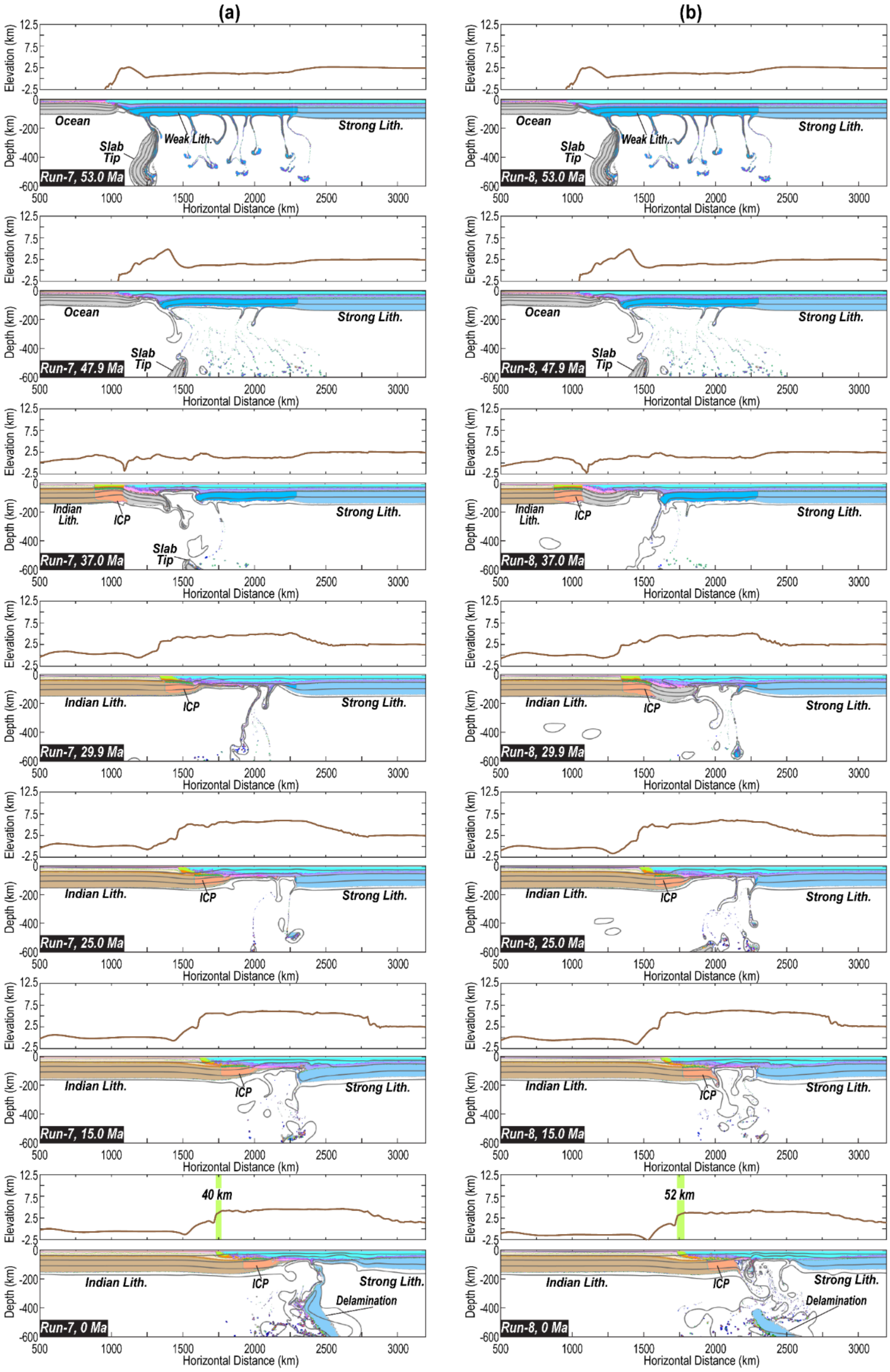

Figure S9. Evolution of material field and surface topography in the Type 3 models. (**a**) Results of Run 7. (**b**) Results of Run 8. (**c**) Results of Run 9. (**d**) Results of Run 10. The green shading with a number

in each bottom panel illustrates the width of the accreted crust (*i.e.*, Tethyan-Greater Himalayas). The compositional density for the ICP lithospheric mantle in each run is: 3.32 g/cm^3, 3.34 g/cm^3, 3.36 g/cm^3, 3.38 g/cm^3, respectively. Other key parameters of these models are in Tables S1-S2. Other plotting habits are the same as those in Figure 5. ICP-Incoming Continental Plate (i.e., Greater India). Lith.- Lithosphere.

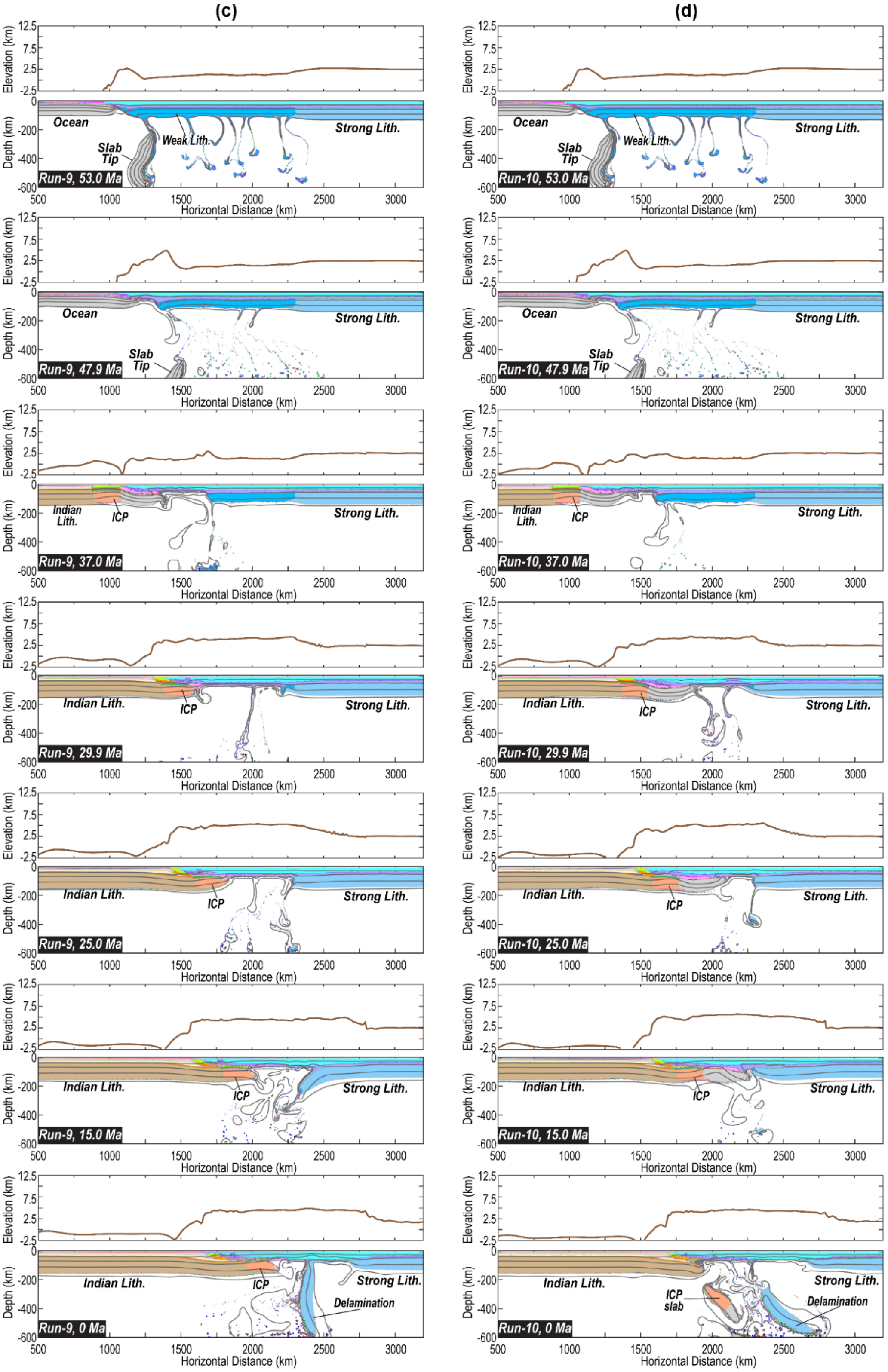

Figure S9 (continued).

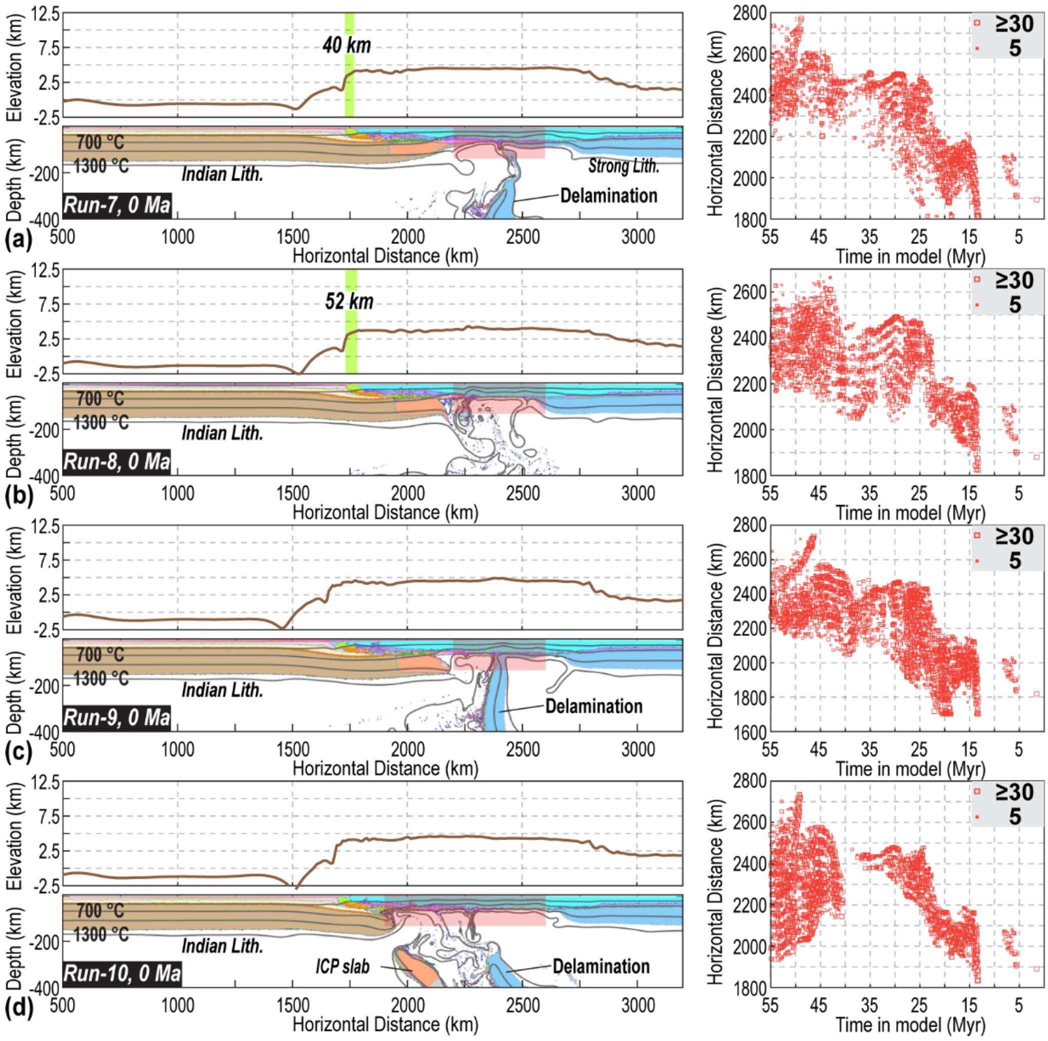

Figure S10. **"Present-day" material field, surface topography, and the melting distribution in the Type 3 models**. (**a**) Results of Run 7. (**b**) Results of Run 8. (**c**) Results of Run 9. (**d**) Results of Run 10. The green shading with a number in each elevation panel illustrates the width of the accreted crust (*i.e.*, Tethyan-Greater Himalayas). The red shading in each material panel highlights the spatial range of melting records. The key parameters of these models are in Tables S1-S2. The plotting habits for melting distribution are the same as those in Figure 7. ICP-Incoming Continental Plate (i.e., Greater India). Lith.-Lithosphere.

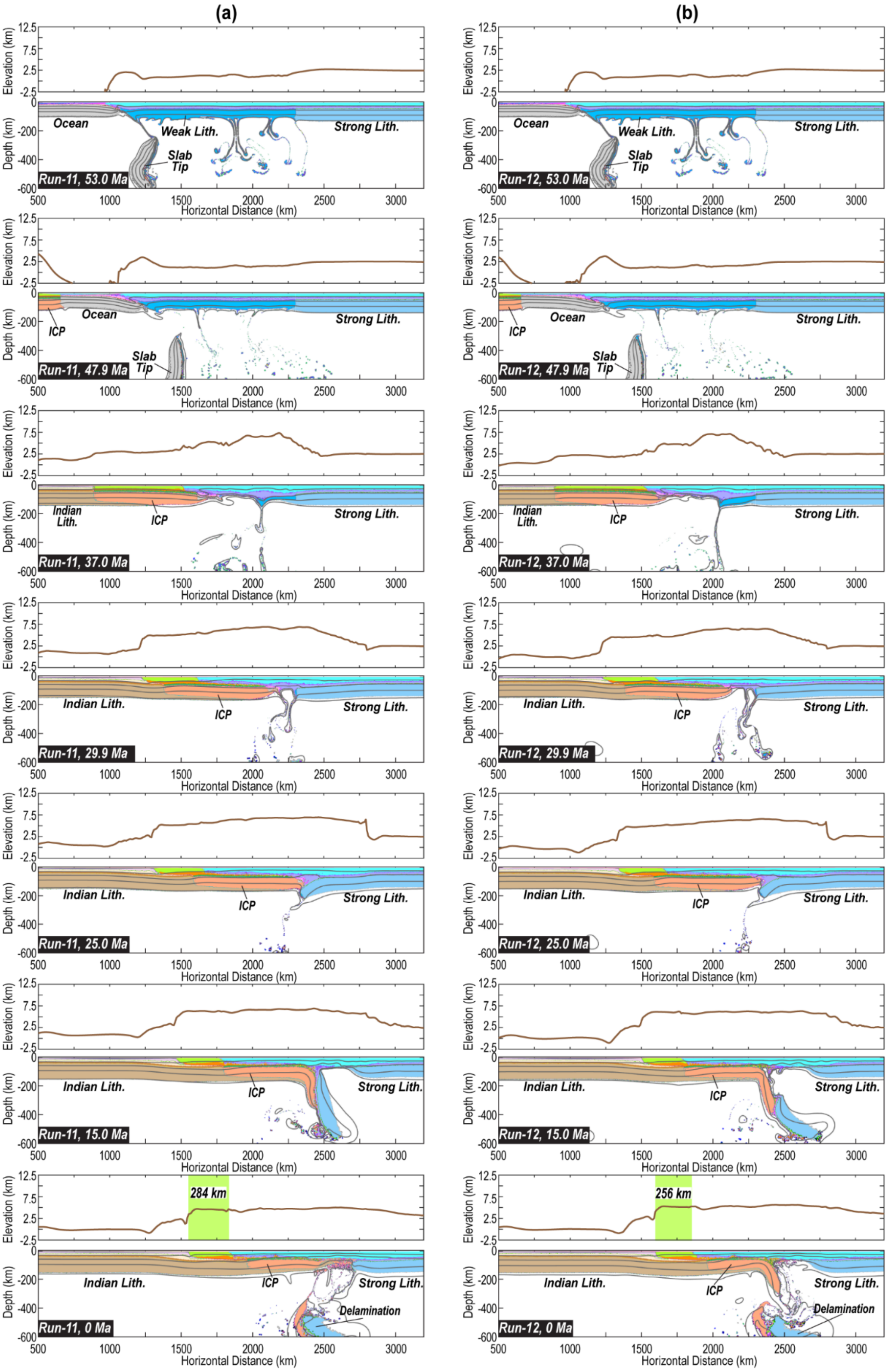

Figure S11. **Evolution of material field and surface topography in the Type 4 models**. (**a**) Results of Run 11. (**b**) Results of Run 12. (**c**) Results of Run 13. (**d**) Results of Run 14. The green shading with a

number in each bottom panel illustrates the accreted crust's width (*i.e.*, Tethyan-Greater Himalayas). The compositional density for the ICP lithospheric mantle in each run is: 3.32 g/cm^3, 3.34 g/cm^3, 3.36 g/cm^3, 3.38 g/cm^3, respectively. Other key parameters of these models are in Tables S1-S2. Other plotting habits are the same as those in Figure 5. ICP-Incoming Continental Plate (i.e., Greater India). Lith.- Lithosphere.

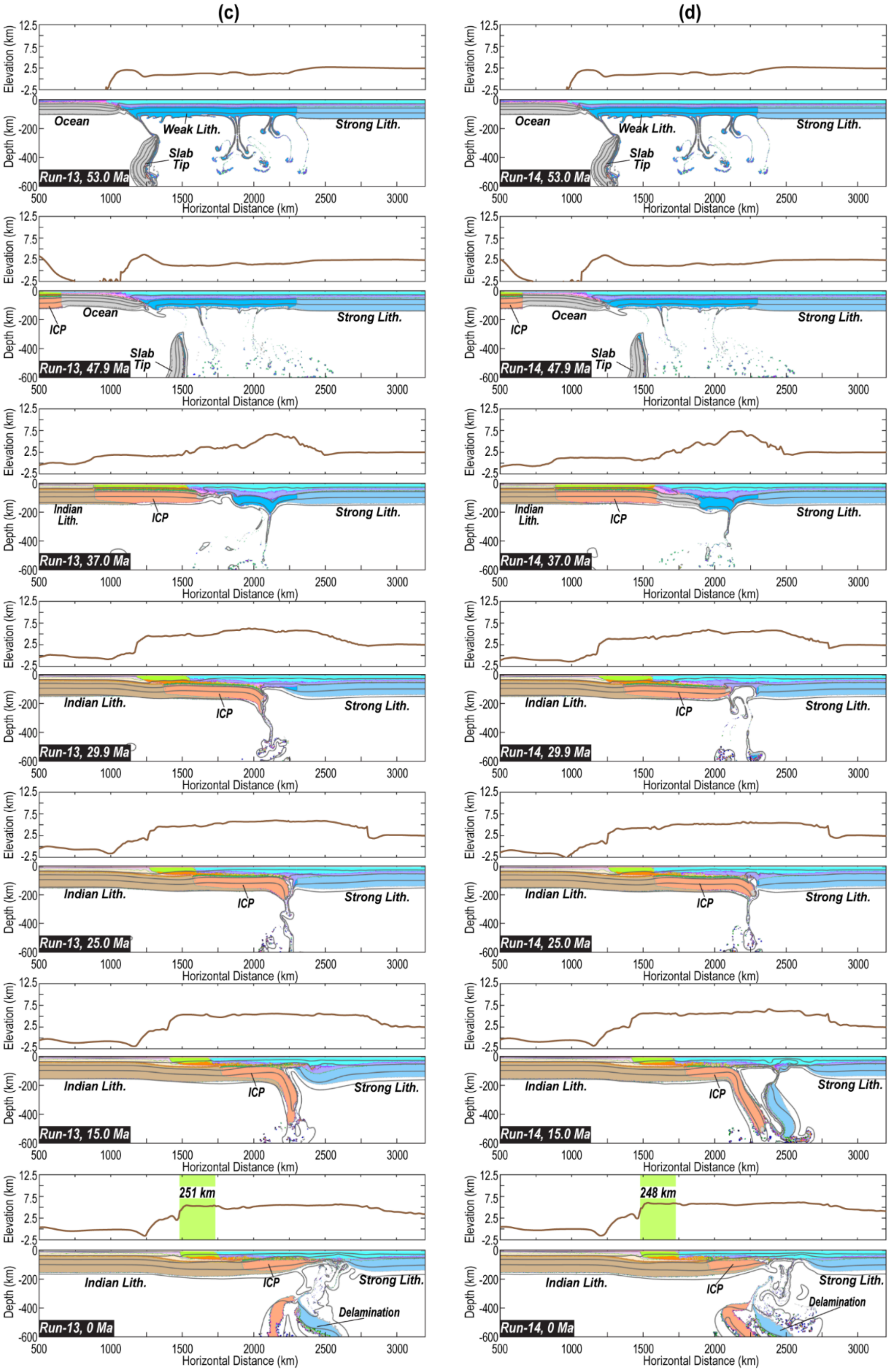

Figure S11 (continued).

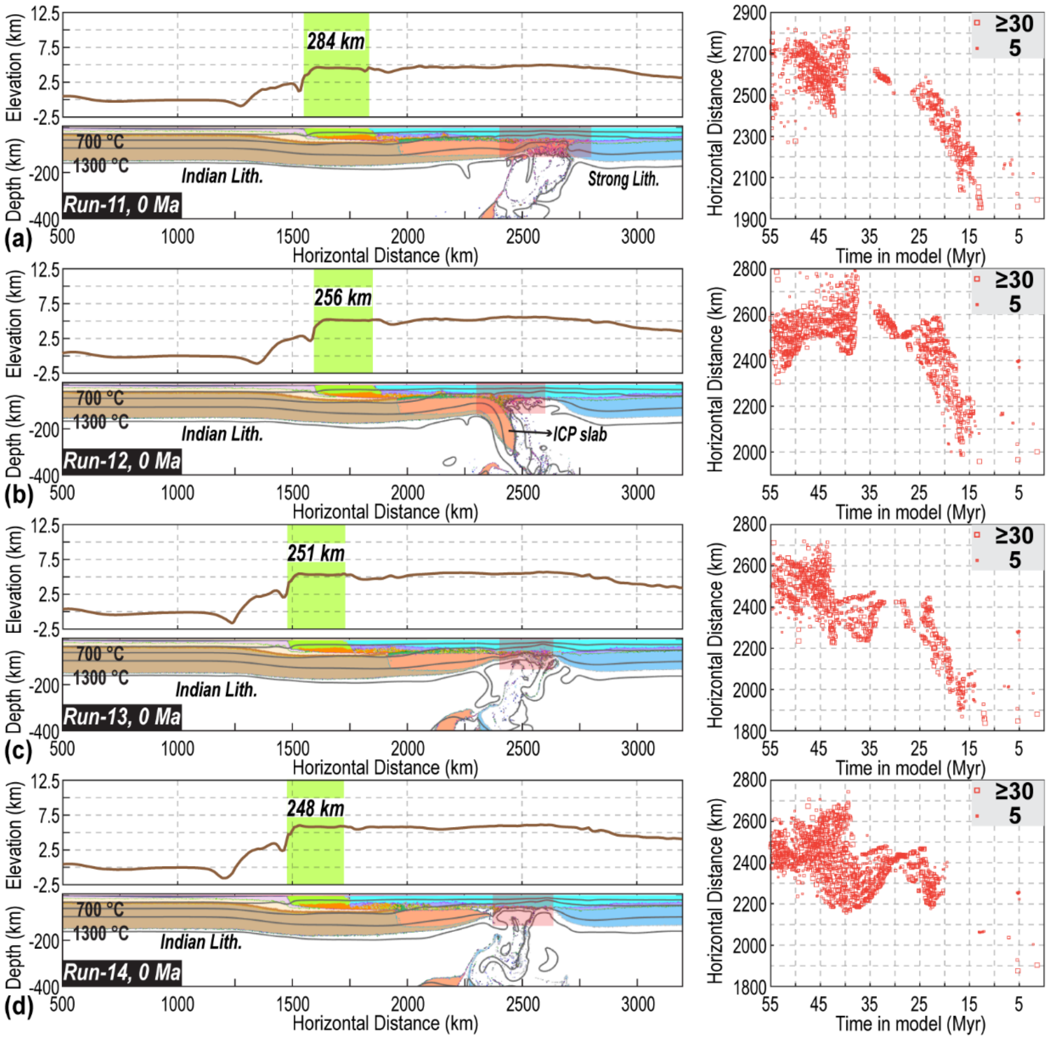

Figure S12. **"Present-day" material field, surface topography, and the melting distribution in the Type 4 models**. (**a**) Results of Run 11. (**b**) Results of Run 12. (**c**) Results of Run 13. (**d**) Results of Run 14. The green shading with a number in each elevation panel illustrates the width of the accreted crust (*i.e.*, Tethyan-Greater Himalayas). The red shading in each material panel highlights the spatial range of melting records. The key parameters of these models are in Tables S1-S2. The plotting habits for melting distribution are the same as those in Figure 7. Lith.- Lithosphere.

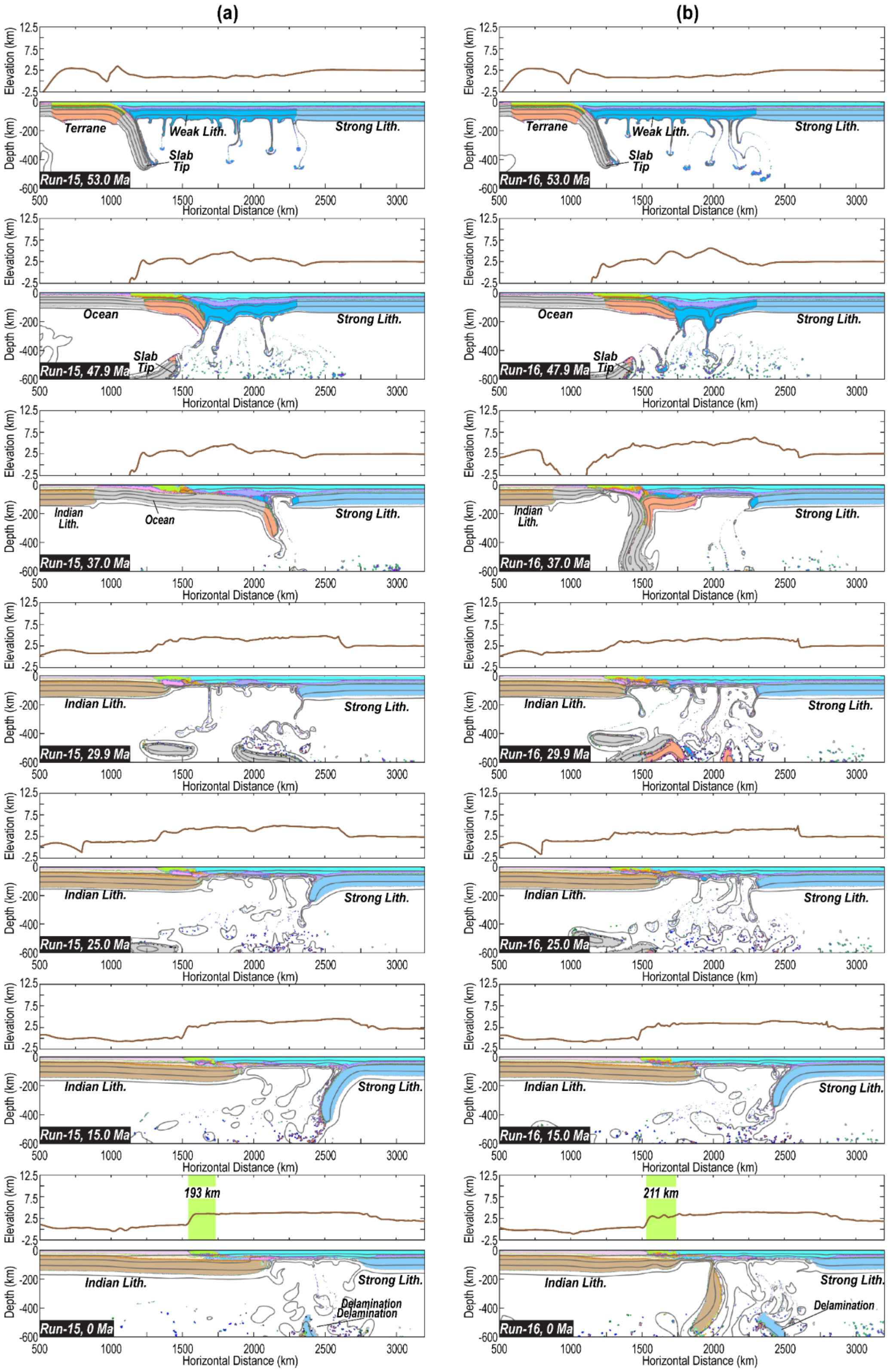

Figure S13. **Evolution of material field and surface topography in the Type 5 models (500 km long terrane)**. (**a**) Results of Run 15. (**b**) Results of Run 16. (**c**) Results of Run 17. (**d**) Results of Run 18. In

these four runs, the terrane is 500 km long. The maximum for weak overriding plate (weak lithosphere) in each run is: 50 MPa, 100 MPa, 150 MPa, 200 Ma, respectively. The green shading with a number in each bottom panel illustrates the accreted crust's width (*i.e.*, Tethyan-Greater Himalayas). Other key parameters of these models are in Tables S1-S2. Other plotting habits are the same as those in Figure 5. Lith.-Lithosphere.

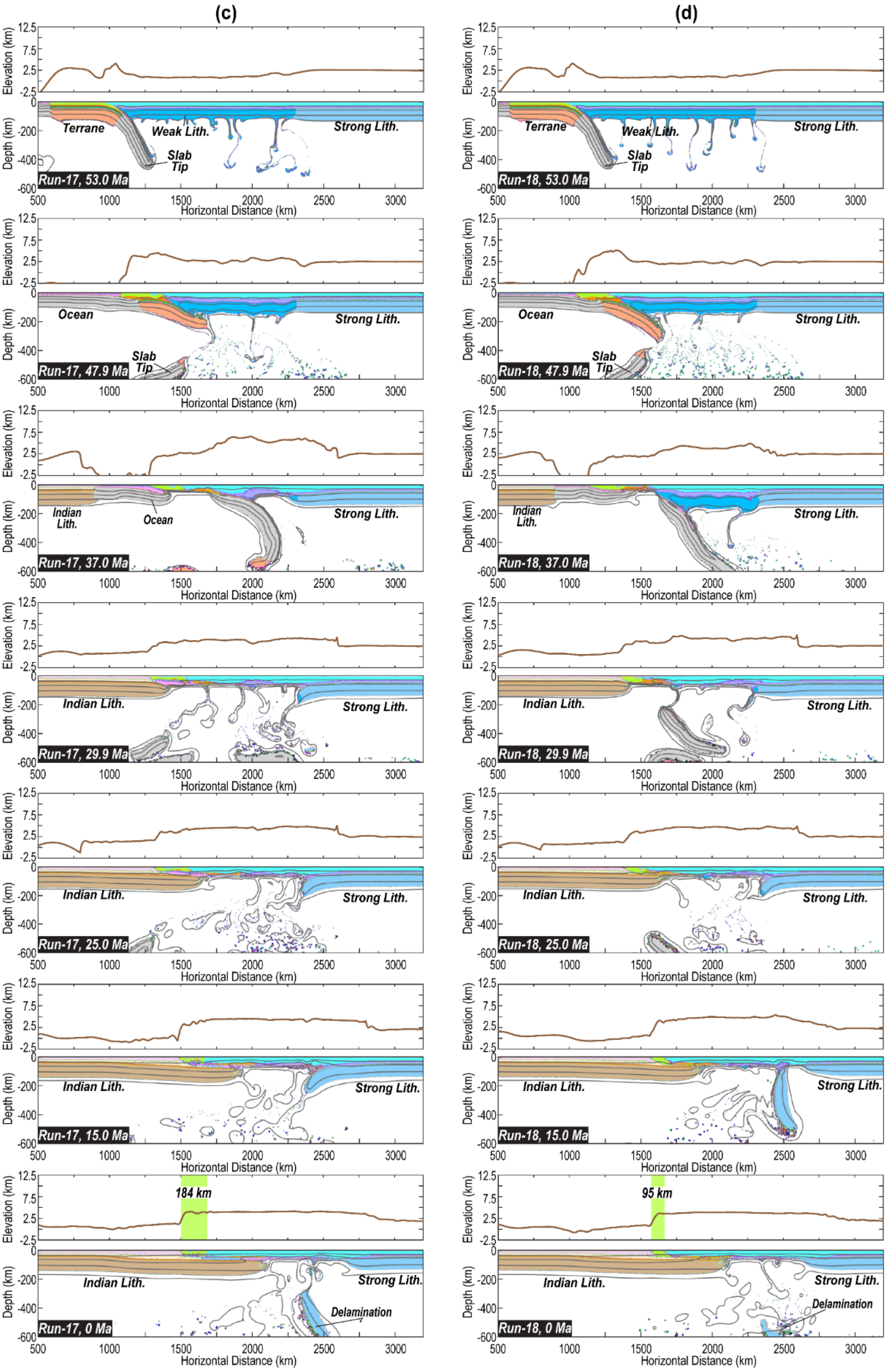

Figure S13 (continued).

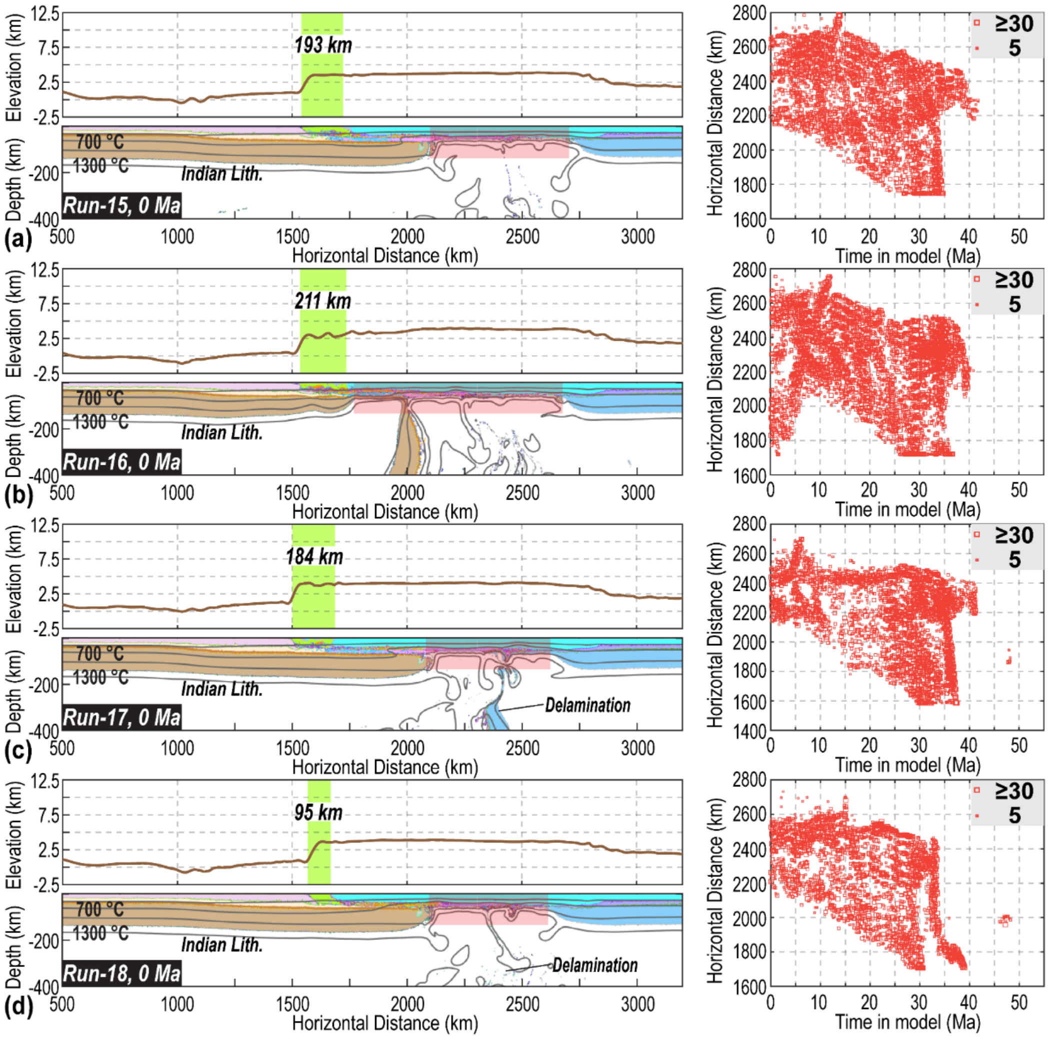

Figure S14. “Present-day” material field, surface topography, and the melting distribution in the Type 5 models. (**a**) Results of Run 15. (**b**) Results of Run 16. (**c**) Results of Run 17. (**d**) Results of Run 18. The green shading with a number in each bottom panel illustrates the accreted crust's width (*i.e.*, Tethyan-Greater Himalayas). The red shading in each material panel highlights the spatial range of melting records. The key parameters of these models are in Tables S1-S2. The plotting habits for melting distribution are the same as those in Figure 7. Lith.- Lithosphere.

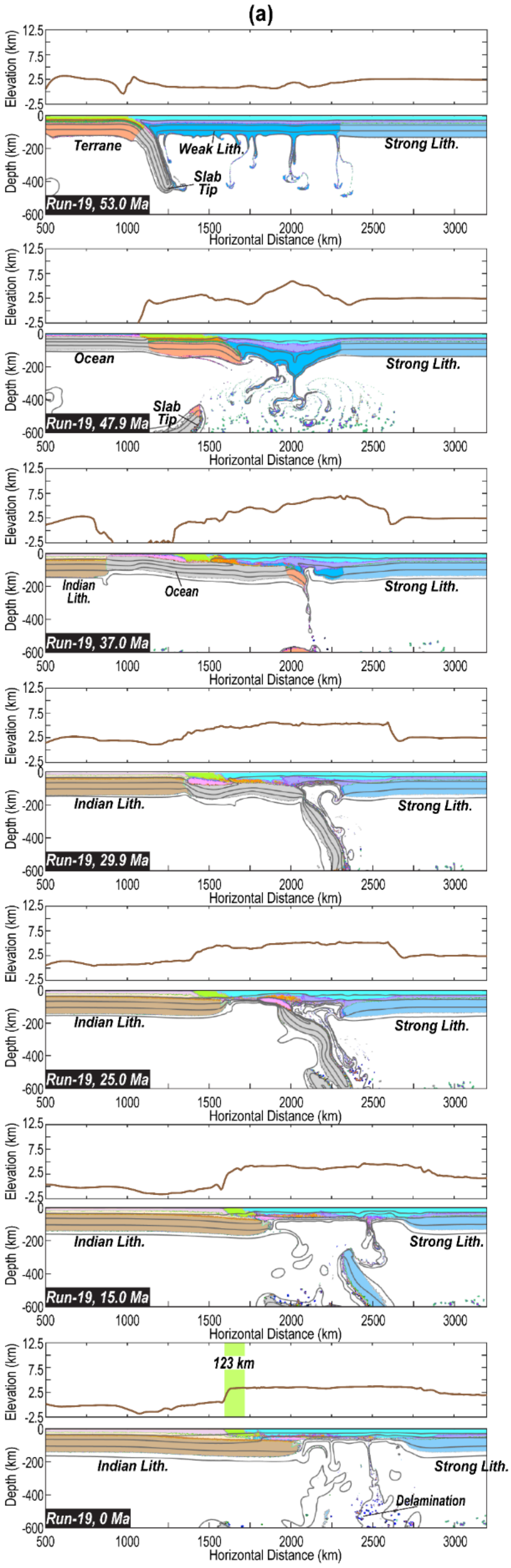

Figure S15. **Evolution of material field and surface topography in the Type 5 models (600 km long terrane)**. (**a**) Results of Run 19. (**b**) Results of Run 20. (**c**) Results of Run 21. (**d**) Results of Run 22. In

these four runs, the terrane is 600 km long. The maximum for weak overriding plate (weak lithosphere) in each run is: 50 MPa, 100 MPa, 150 MPa, 200 Ma, respectively. The green shading with a number in each bottom panel illustrates the accreted crust's width (*i.e.*, Tethyan-Greater Himalayas). Other key parameters of these models are in Tables S1-S2. Other plotting habits are the same as those in Figure 5. Lith.- Lithosphere.

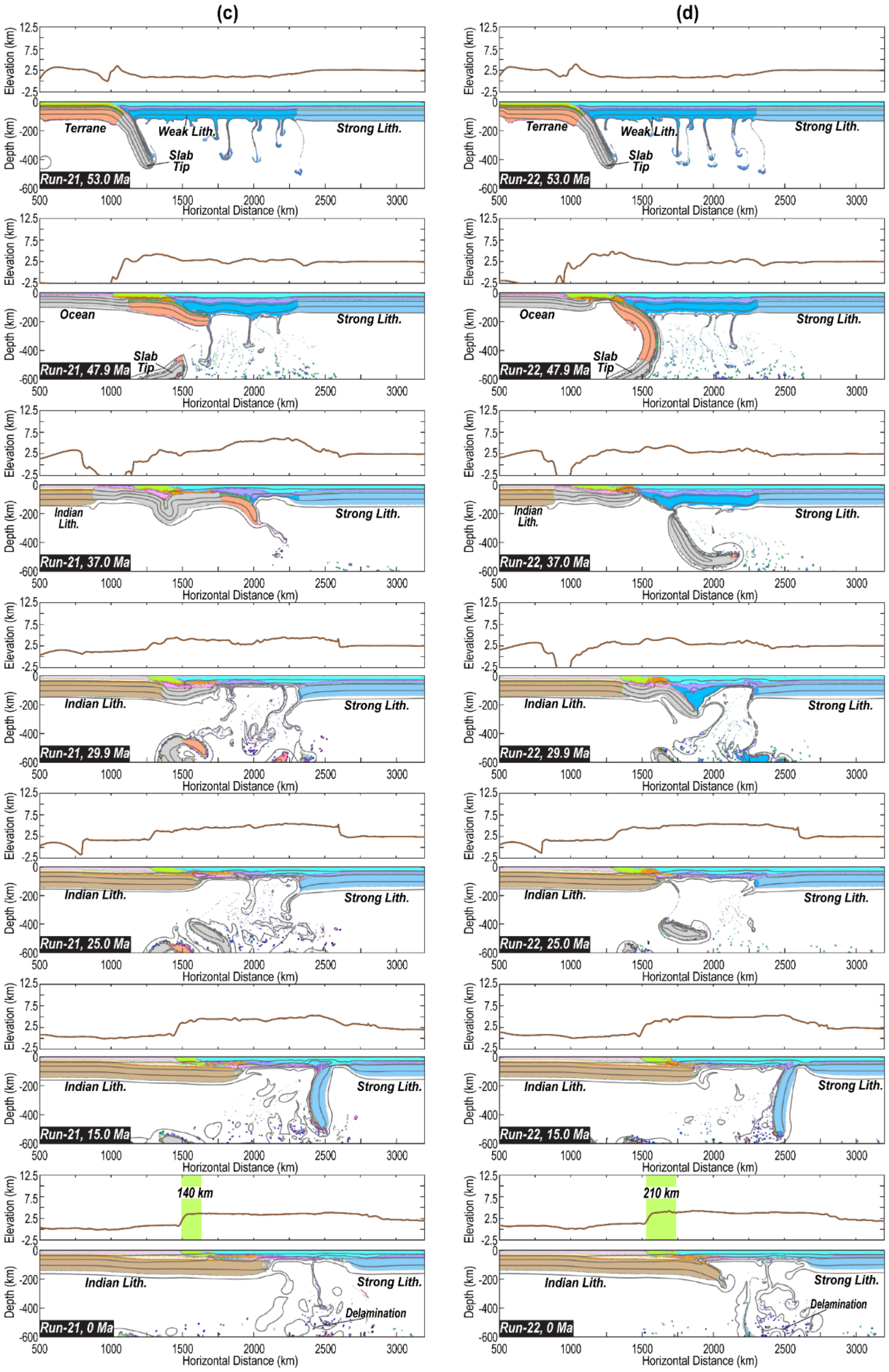

Figure S15 (continued).

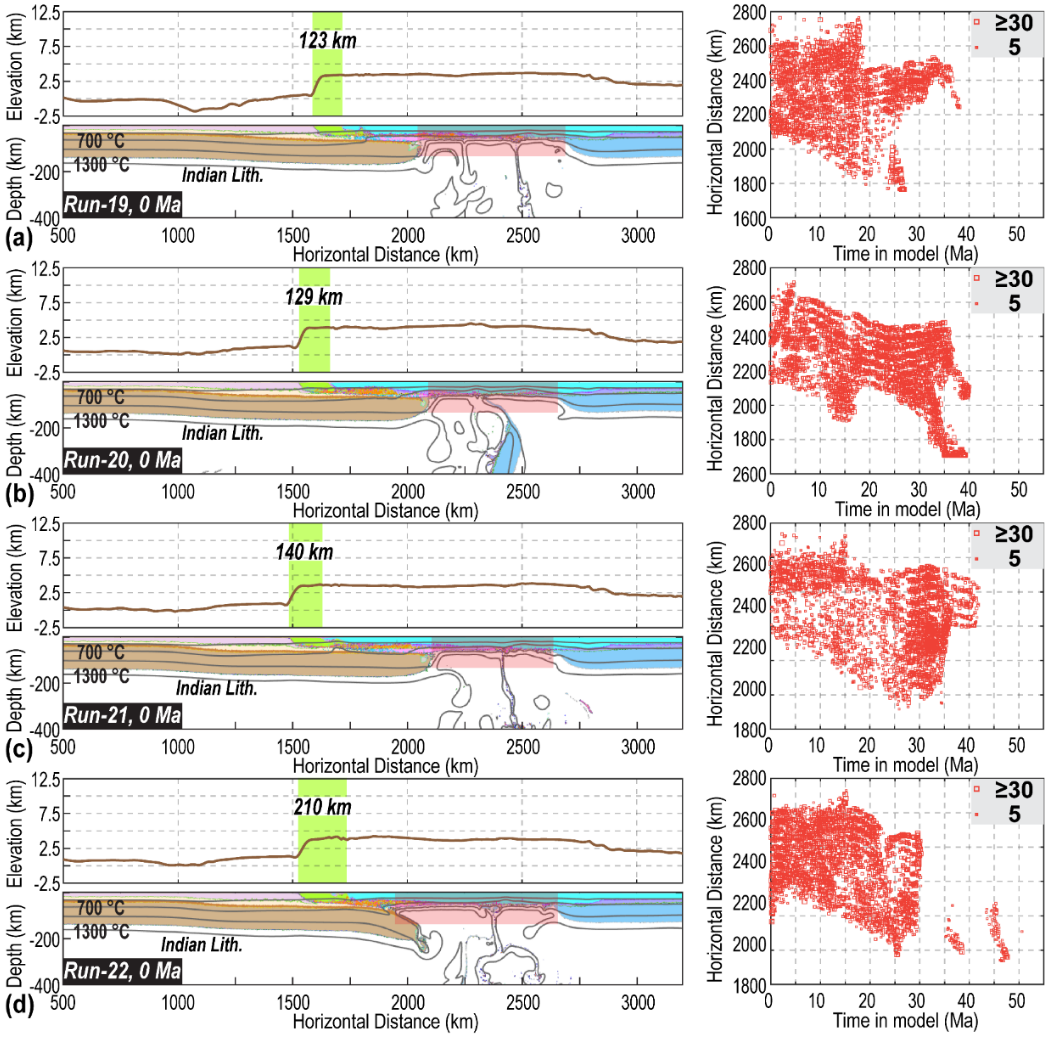

Figure S16. "Present-day" material field, surface topography, and the melting distribution in the Type 5 models. (**a**) Results of Run 19. (**b**) Results of Run 20. (**c**) Results of Run 21. (**d**) Results of Run 22. The green shading with a number in each bottom panel illustrates the accreted crust's width (*i.e.*, Tethyan-Greater Himalayas). The red shading in each material panel highlights the spatial range of melting records. The key parameters of these models are in Tables S1-S2. The plotting habits for melting distribution are the same as those in Figure 7. Lith.- Lithosphere.

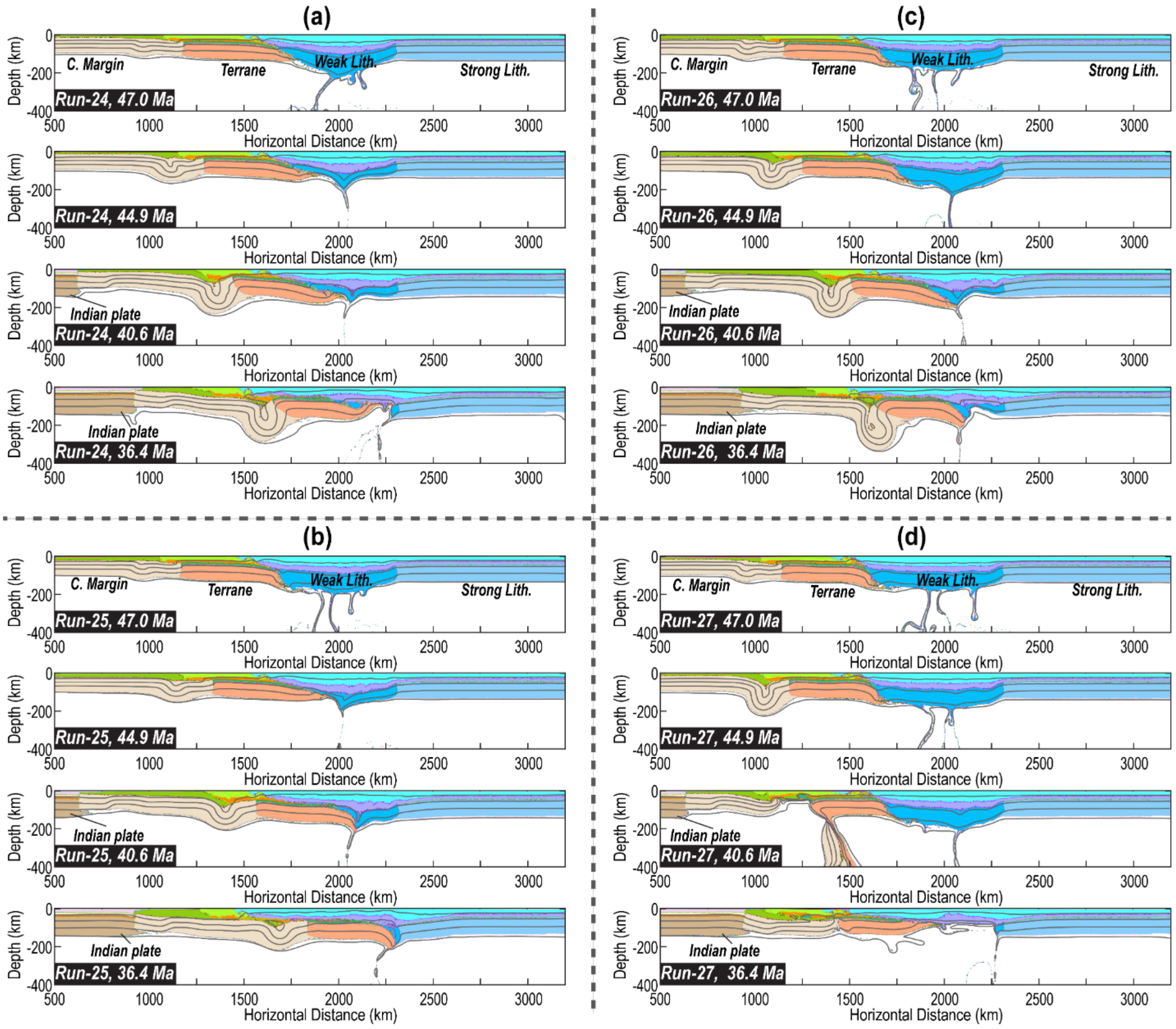

Figure S17. **Effects of overriding-plate rheology on the evolution of the Type 6 model**. (**a**) Results of Run 24. This model is the same as Run 23, except that the maximum yielding stress for the weak overriding plate (Tibetan terranes) crust is capped at 100 Mpa. (**b**) Results of Run 25. This model is the same as Run 23, except that the maximum yielding stress for the weak overriding plate (Tibetan terranes) crust is capped at 150 Mpa. (**c**) Results of Run 26. This model is the same as Run 23, except that the maximum yielding stress for the weak overriding plate (Tibetan terranes) crust is capped at 200 Mpa. (**d**) Results of Run 27. This model is the same as Run 23, except that the maximum yielding stress for the lithospheric mantle of the weak overriding plate (Tibetan terranes) is capped at 100 Mpa. More parameters are in Tables S1-S2. Lith.- Lithosphere. C. Margin- Continental Margin.

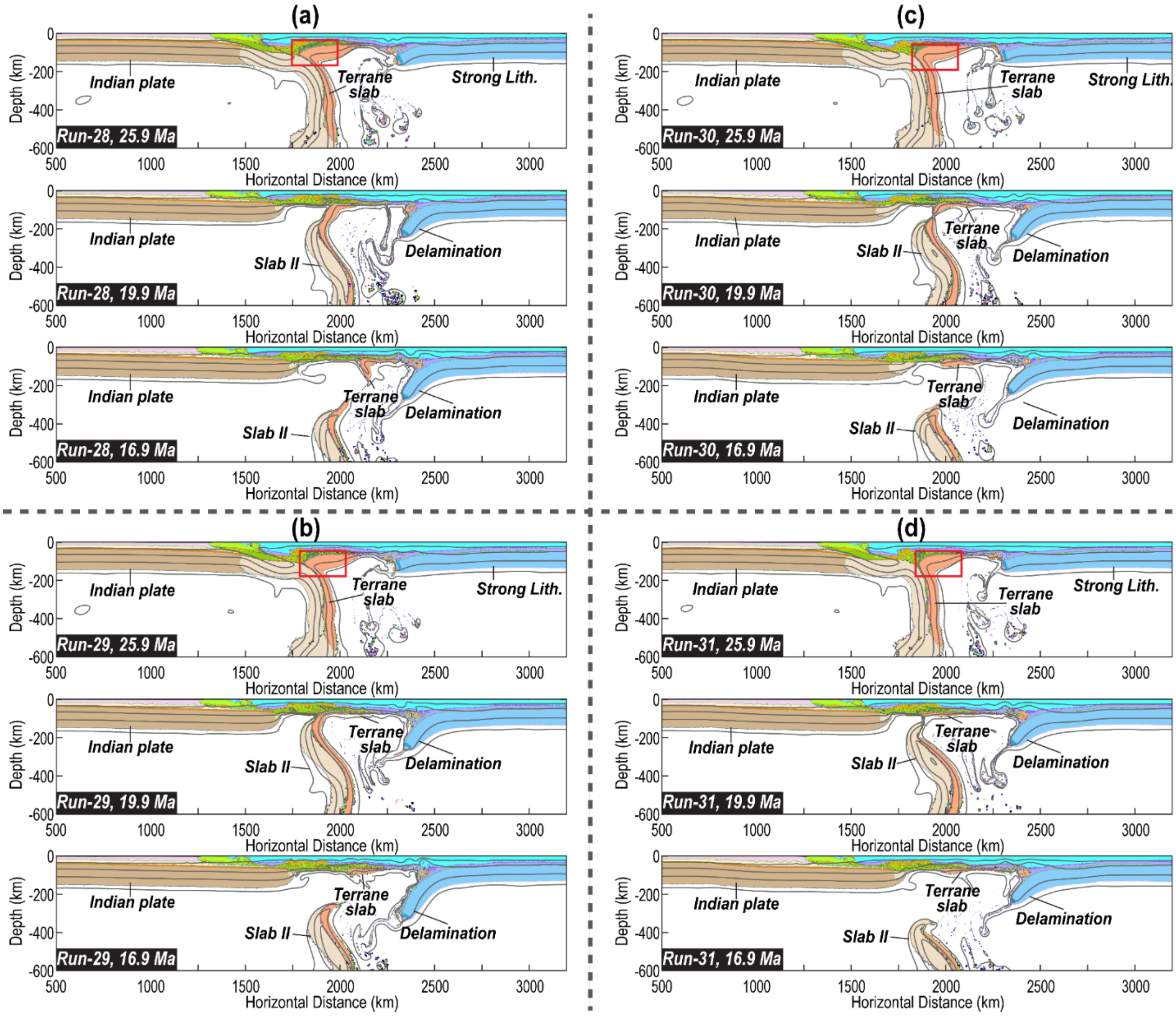

Figure S18. **Effects of terrane slab rheology on the evolution of the Type 6 model**. (**a**) Results of Run 28. This model is the same as Run 23, except that the maximum yielding stress for the terrane slab hinge is capped at 40 Mpa. Terrane slab hinge (within the red box) refers to the terrane portion above the oceanic slab. (**b**) Results of Run 29. This model is the same as Run 23, except that the maximum yielding stress for the terrane slab hinge is capped at 60 Mpa. (**c**) Results of Run 30. This model is the same as Run 23, except that the maximum yielding stress for the terrane slab hinge is capped at 80 Mpa. (**d**) Results of Run 31. This model is the same as Run 23, except that the maximum yielding stress for the terrane slab hinge is capped at 100 Mpa. More parameters are in Tables S1-S2. Lith.- Lithosphere.

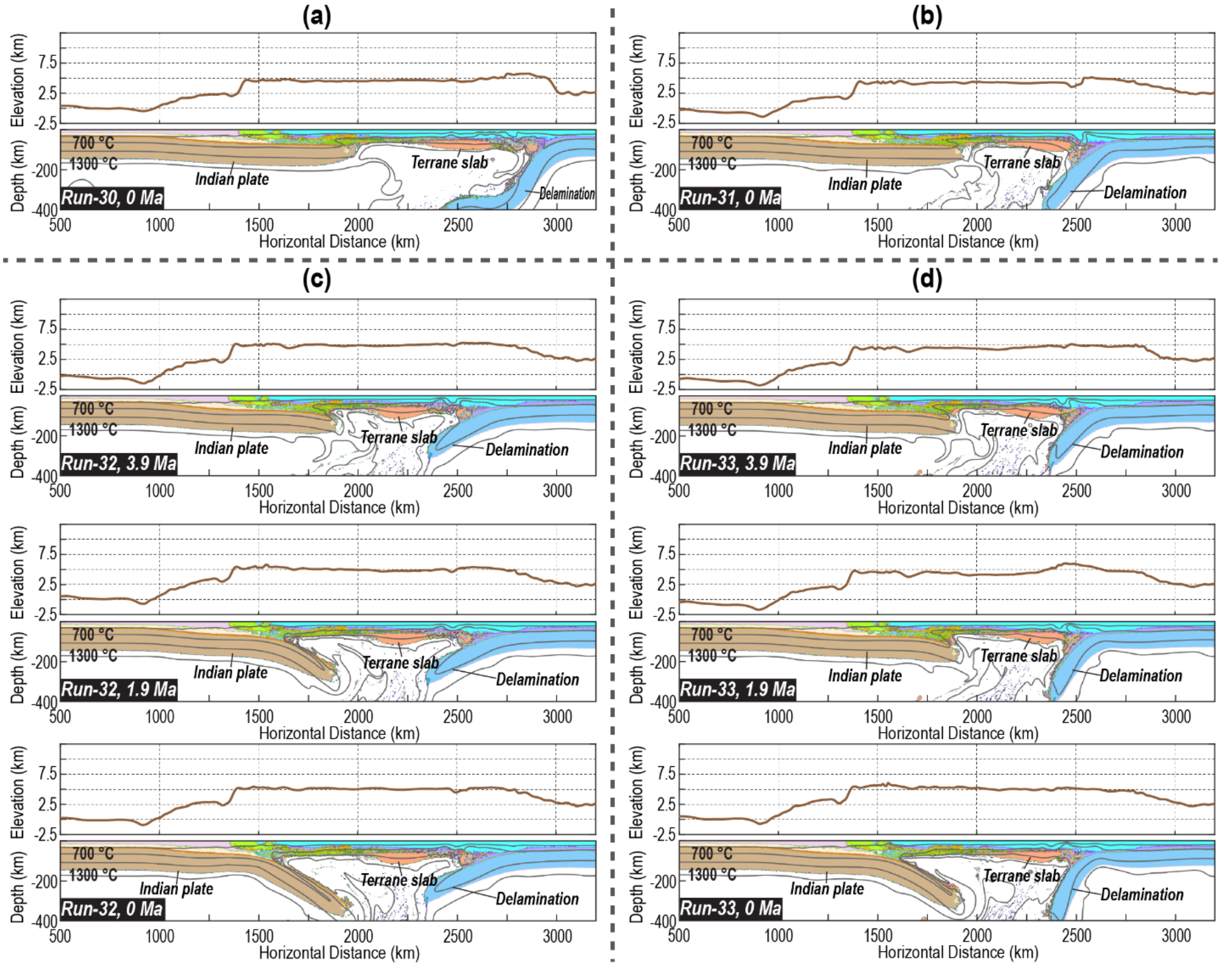

Figure S19. **Effects of the Indian plate density on the evolution of the Type 6 model**. (**a**) Results of Run 32. This model is the same as Run 23, except that the compositional density of the Indian lithospheric mantle is 3.35 g/cm^3. (**b**) Results of Run 33. This model is the same as Run 23, except that the compositional density of the Indian lithospheric mantle is 3.36 g/cm^3. (**c**) Results of Run 34. This model is the same as Run 23, except that the compositional density of the Indian lithospheric mantle is 3.37 g/cm^3. (**d**) Results of Run 35. This model is the same as Run 23, except that the compositional density of the Indian lithospheric mantle is 3.38 g/cm^3. More parameters are in Tables S1-S2. Lith.- Lithosphere.

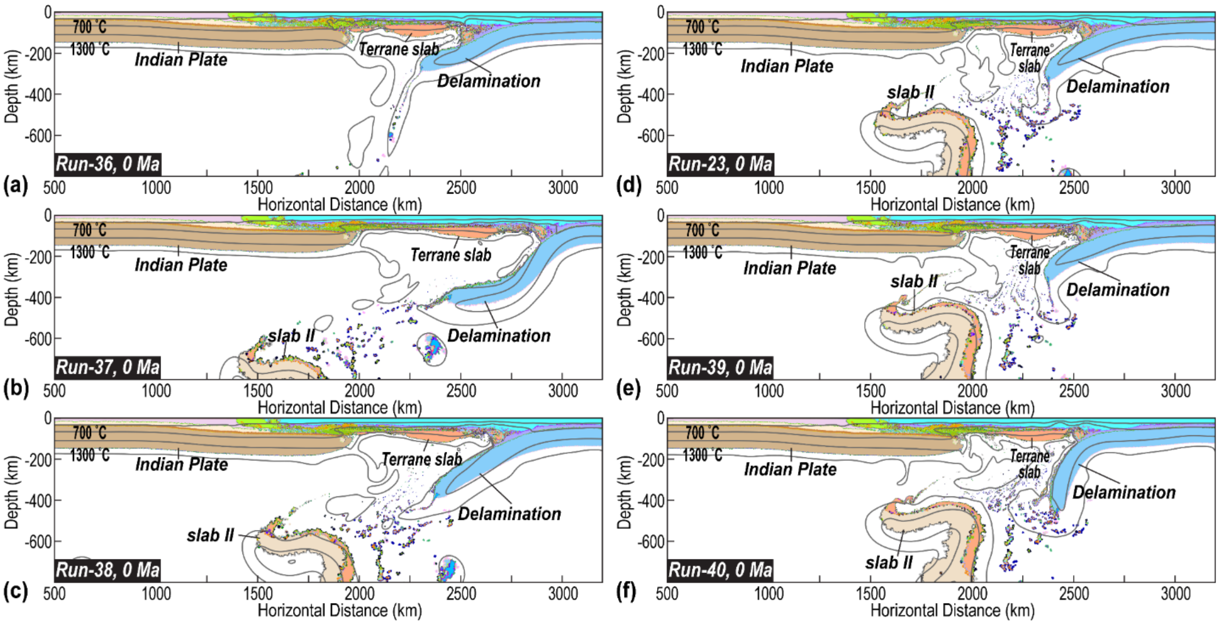

Figure S20. **Effects of the lower mantle viscosity on the evolution of the Type 6 model**. (**a**) Results of Run 36. This model is the same as Run 23, except for the lower mantle viscosity of 10^{22} Pa·s. (**b**) Results of Run 37. This model is the same as Run 23, except that the lower mantle viscosity is 3×10^{22} Pa·s. (**c**) Results of Run 38. This model is the same as Run 23, except that the lower mantle viscosity is 5×10^{22} Pa·s. (**d**) Results of Run 23. In Run 23, the lower mantle viscosity is 10×10^{22} Pa·s. **(e)** Results of Run 39. This model is the same as Run 23, except that the lower mantle viscosity is 20×10^{22} Pa·s. (**f**) Results of Run 40. This model is the same as Run 23, except that the lower mantle viscosity is 50×10^{22} Pa·s. More parameters are in Tables S1-S2. Lith.- Lithosphere.

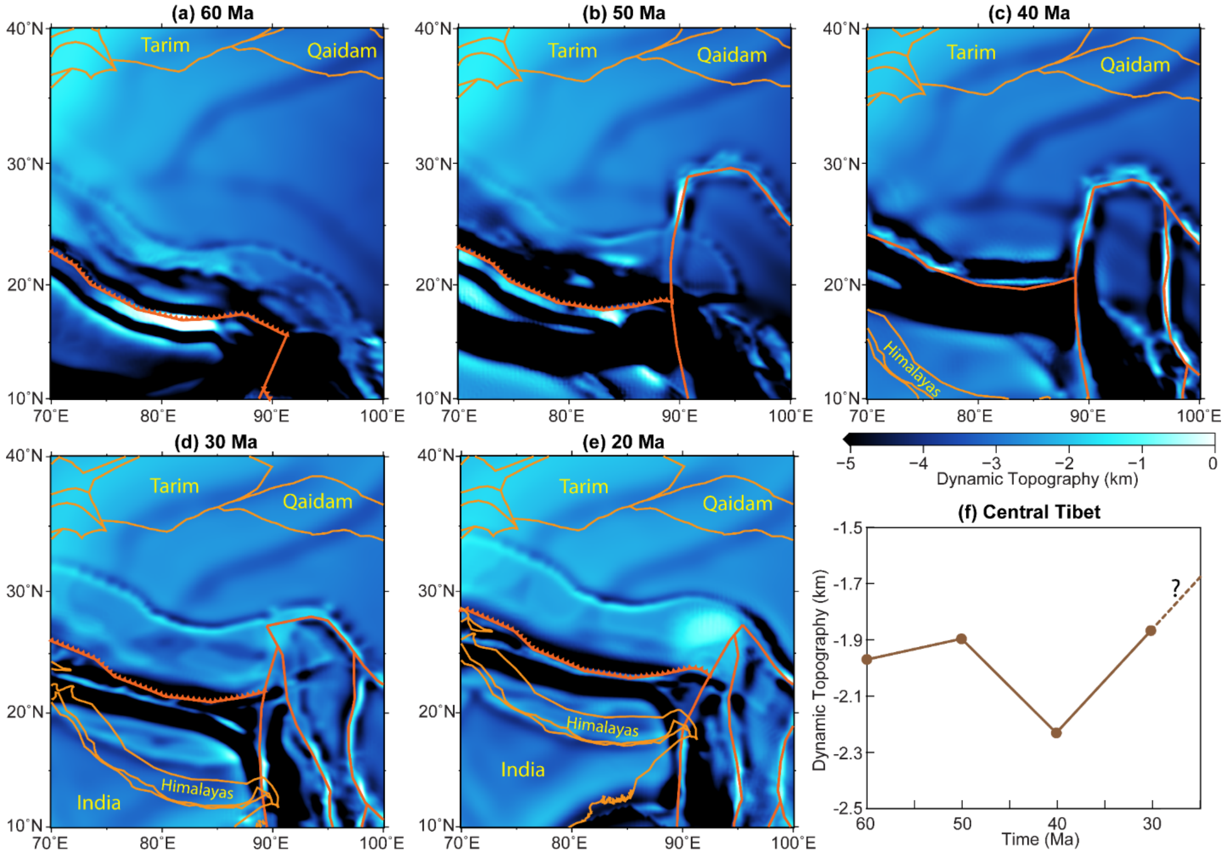

Figure S21. Dynamic topography evolution of the Indo-Eurasian Orogen according to the global numerical modeling[17]. The distribution of faults, sutures, and trenches is based on Müller et al., 2016[18]. **(a)-(e)** Map views of the dynamic topography at 60, 50, 40, 30, and 20 Ma, respectively. **(f)** Evolution of the dynamic topography in central Tibet. Because the Indian Subcontinental is our 3D model is purely thermal, the dynamic topography of the Tibet Plateau after the India approach can be too large [19-20].

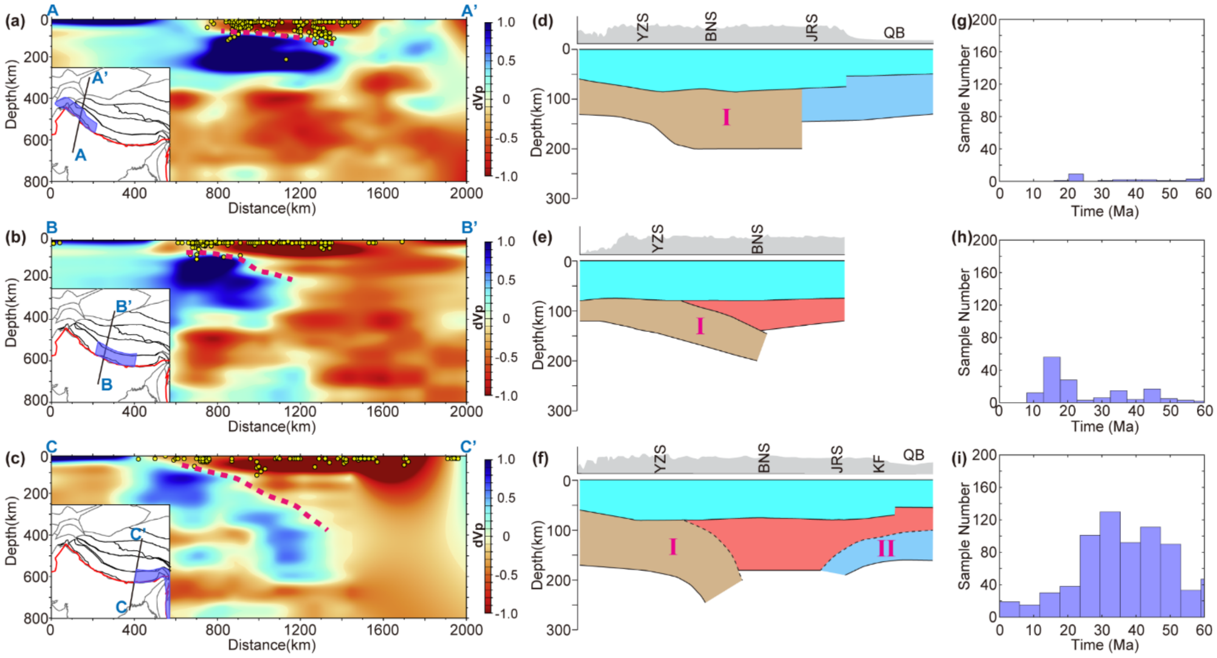

Figure S22. (a)-(c) The P-wave seismic tomography images of the upper mantle beneath North India and Tibet Plateau (after Li et al., 2008)[21]. **(d)-(f)** Sketch illustrations of the upper mantle structure beneath the Tibet Plateau based on P and S receiver function[22] along similar locations in **a-c**. **(g)-(i)** The number of magma samples in regions close to the suture[23,24], with the purple shadings in **a-c** demonstrating the sample locations.

Supplementary References:

1 Liu, L., Liu, L. & Xu, Y. G. Intermittent Post-Paleocene Continental Collision in South Asia. *Geophysical Research Letters* **48**, e2021GL094531 (2021a).

2 Liu, L., Liu, L. & Xu, Y.-G. Mesozoic intraplate tectonism of East Asia due to flat subduction of a composite terrane slab. *Earth-Science Reviews* **214**, 103505 (2021b).

3 Hirth, G. & Kohlstedt, D. Rheology of the upper mantle and the mantle wedge: A view from the experimentalists. *Geophysical monograph-american geophysical union* **138**, 83-106 (2003).

4 Ghosh, A. & Holt, W. E. Plate motions and stresses from global dynamic models. *Science* **335**, 838-843 (2012).

5 Singh, A. *et al.* Crustal structure beneath India and Tibet: New constraints from inversion of receiver functions. *Journal of Geophysical Research: Solid Earth* **122**, 7839-7859 (2017).

6 Brune, S., Heine, C., Pérez-Gussinyé, M. & Sobolev, S. V. Rift migration explains continental margin asymmetry and crustal hyper-extension. *Nature communications* **5**, 1-9 (2014).

7 Gleason, G. C. & Tullis, J. A flow law for dislocation creep of quartz aggregates determined with the molten salt cell. *Tectonophysics* **247**, 1-23 (1995).

8 Rutter, E. & Brodie, K. Experimental grain size-sensitive flow of hot-pressed Brazilian quartz aggregates. *Journal of Structural Geology* **26**, 2011-2023 (2004).

9 Kirby, S. & Kronenberg, A. Rheology of the lithosphere: Selected topics. *Reviews of Geophysics* **25**, 1219-1244 (1987).

10 Ranalli, G. & Murphy, D. C. Rheological stratification of the lithosphere. *Tectonophysics* **132**, 281-295 (1987).

11 Huangfu, P. *et al.* Multi-terrane structure controls the contrasting lithospheric evolution beneath the western and central–eastern Tibetan plateau. *Nature Communications* **9**, 1-11 (2018).

12 Rybacki, E. & Dresen, G. Deformation mechanism maps for feldspar rocks. *Tectonophysics* **382**, 173-187 (2004).

13 Djomani, Y. H. P., O'Reilly, S. Y., Griffin, W. & Morgan, P. The density structure of subcontinental lithosphere through time. *Earth and Planetary Science Letters* **184**, 605-621 (2001).

14 Schutt, D. & Lesher, C. Effects of melt depletion on the density and seismic velocity of garnet and spinel lherzolite. *Journal of Geophysical Research: Solid Earth* **111** (2006).

15 Karato, S.-i. & Wu, P. Rheology of the upper mantle: A synthesis. *Science* **260**, 771-778 (1993).

16 Yamazaki, D. & Karato, S.-i. Some mineral physics constraints on the rheology and geothermal structure of Earth's lower mantle. *American Mineralogist* **86**, 385-391 (2001).

17 Peng, D., Liu, L., Hu, J., Li, S. & Liu, Y. Formation of East Asian Stagnant Slabs Due To a Pressure-Driven Cenozoic Mantle Wind Following Mesozoic Subduction. *Geophysical Research Letters* **48**, e2021GL094638 (2021).

18 Müller, R. D. *et al.* Ocean basin evolution and global-scale plate reorganization events since Pangea breakup. *Annual Review of Earth and Planetary Sciences* **44**, 107-138 (2016).

19 Müller, R., Hassan, R., Gurnis, M., Flament, N. & Williams, S. E. Dynamic topography of passive continental margins and their hinterlands since the Cretaceous. *Gondwana Research* **53**, 225-251 (2018).

20 Husson, L. *et al.* Dynamic ups and downs of the Himalaya. *Geology* **42**, 839-842 (2014).

21 Li, C., Van der Hilst, R. D., Meltzer, A. S. & Engdahl, E. R. Subduction of the Indian lithosphere beneath the Tibetan Plateau and Burma. *Earth and Planetary Science Letters* **274**, 157-168 (2008).

22 Zhao, J. *et al.* The boundary between the Indian and Asian tectonic plates below Tibet. *Proceedings of the National Academy of Sciences* **107**, 11229-11233 (2010).

23 Yakovlev, P. V. *et al.* The geochemistry of Tibetan lavas: Spatial and temporal relationships, tectonic links and geodynamic implications. *Earth and Planetary Science Letters* **520**, 115-126 (2019).

24 Deng, J., Wang, Q., Li, G. & Santosh, M. Cenozoic tectono-magmatic and metallogenic processes in the Sanjiang region, southwestern China. *Earth-Science Reviews* **138**, 268-299 (2014).
