# Supplementary material for: New constraints on Cenozoic subduction between India and Tibet
Source: Nat Commun. 2023 Apr 7;14:1963. doi: 10.1038/s41467-023-37615-5 (PMC10082029; doi:10.1038/s41467-023-37615-5)
Supplement: Supplementary file 2 — Description of Additional Supplementary files [file 41467_2023_37615_MOESM2_ESM.pdf]

Description of Additional Supplementary Files

Seven movies highlight and summarize the range of model evolutions (Figure 1b), using the same plotting conventions as those in Figure 5) (Table S2). An additional movie shows the time evolution of viscosity and density fields in our preferred Type 6 model (Run 23).

Supplementary Movie-1: Type 1 Model (Run 4)

Supplementary Movie-2: Type 2 Model (Run 6)

Supplementary Movie-3: Type 3 model (Run 9)

Supplementary Movie-4: Type 4 model (Run 13)

Supplementary Movie-5: Type 5 Model (Run 15)

Supplementary Movie-6: Type 5 Model (Run 18)

Supplementary Movie-7: Type 6 Model (Run 23)

Supplementary Movie-8: Evolution of viscosity and density fields in the Type 6 model (Run 23). Here plotting conventions are the same as those in Figures S1 and S4.
